# Advanced Design Concepts for Shape-Memory Polymers in Biomedical Applications and Soft Robotics

**DOI:** 10.3390/polym18020214

**Published:** 2026-01-13

**Authors:** Anastasia A. Fetisova, Maria A. Surmeneva, Roman A. Surmenev

**Affiliations:** 1 Physical Materials Science and Composite Materials Centre, Research School of Chemistry and Applied Biomedical Sciences, National Research Tomsk Polytechnic University, 30 Lenina Avenue, Tomsk 634050, Russia; aaf71@tpu.ru (A.A.F.); surmenevamaria@tpu.ru (M.A.S.); 2 International Research and Development Centre Piezo- and Magnetoelectric Materials, Research School of Chemistry and Applied Biomedical Sciences, National Research Tomsk Polytechnic University, 30 Lenina Avenue, Tomsk 634050, Russia

**Keywords:** shape-memory polymers, biodegradable polymers, regenerative medicine, additive manufacturing

## Abstract

Shape-memory polymers (SMPs) are a class of smart materials capable of recovering their original shape from a programmed temporary shape in response to external stimuli such as heat, light, or magnetic fields. SMPs have attracted significant interest for biomedical devices and soft robotics due to their large recoverable strains, programmable mechanical and thermal properties, tunable activation temperatures, responsiveness to various stimuli, low density, and ease of processing via additive manufacturing techniques, as well as demonstrated biocompatibility and potential bioresorbability. This review summarises recent progress in the fundamentals, classification, activation mechanisms, and fabrication strategies of SMPs, focusing particularly on design principles that influence performance relevant to specific applications. Both thermally and non-thermally activated SMP systems are discussed, alongside methods for controlling activation temperatures, including plasticisation, copolymerisation, and modulation of cross-linking density. The use of functional nanofillers to enhance thermal and electrical conductivity, mechanical strength, and actuation efficiency is also considered. Current manufacturing techniques are critically evaluated in terms of resolution, material compatibility, scalability, and integration potential. Biodegradable SMPs are highlighted, with discussion of degradation behaviour, biocompatibility, and demonstrations in devices such as haemostatic foams, embolic implants, and bone scaffolds. However, despite their promising potential, the widespread application of SMPs faces several challenges, including non-uniform activation, the need to balance mechanical strength with shape recovery, and limited standardisation. Addressing these issues is critical for advancing SMPs from laboratory research to clinical and industrial applications.

## 1. Introduction

In recent years, rapid progress in biomedicine, soft robotics, and aerospace technologies has created an urgent demand for multifunctional, highly adaptable smart materials. Shape-memory polymers (SMPs) have attracted significant attention because of their ability to recover a programmed temporary shape when exposed to external stimuli. Common external stimuli include temperature [1,2], light [3], pH [4], ultrasound [5], microwave radiation [6], alternating magnetic fields [7], electricity [8], ions [9], enzymes [10], solvents [11,12], and others [13]. This makes SMPs highly versatile for a range of applications, from medical implants to soft actuators.

SMPs can temporarily fix a deformed shape through a programmed shape-memory process. In this process, the SMP is heated above the transition temperature (*T*_trans_; glass transition temperature *T*_g_ or melting temperature *T*_m_), deformed, fixed in a temporary shape under an applied load, and then cooled below *T*_trans_. Upon exposure to heat, the SMP recovers its original shape. This shape recovery occurs as the polymer segments spontaneously reorganise towards a thermodynamically preferred, high-entropy state.

Unlike shape-memory alloys (SMAs), where the shape-memory effect (SME) arises from martensitic–austenitic phase transformations, SMPs depend on polymer chain mobility. They offer several advantages over SMAs (NiTi [14], CuAlNi [15], and FeMnSi [16]), including low density, simple processing, low cost, biocompatibility, biodegradability, and a high recoverable strain of up to 300% [17]. SMPs can be made using traditional methods like extrusion and moulding, as well as more modern techniques like electrospinning and additive manufacturing [18]. These characteristics make SMPs a competitive substitute for SMAs in many engineering applications.

Emerging manufacturing techniques are broadening the design possibilities for SMPs. Electrospinning produces ultra-thin fibres with precisely controlled morphology and a high specific surface area, essential for SMPs that require rapid response and enhanced mechanical performance [19]. In addition, 3D printing technologies enable the precise fabrication of complex geometries from digital models. Common technologies for SMPs include fused deposition modelling (FDM), fused filament fabrication (FFF), direct ink writing (DIW) [20], digital light processing (DLP) [21], and two-photon polymerisation (TPP) [22]. These technologies are used to manufacture materials that demonstrate high shape accuracy, improved mechanical properties [23] and shape-memory performance [24]. They often outperform conventionally moulded or cast samples [25]. Notably, TPP lithography enables the fabrication of submicron structures with a resolution up to 300 nm, which can recover their original shape within seconds after being heated above the SMP’s *T*_g_ [26].

Nowadays, SMPs are used in a variety of fields. In biomedicine, for example, they enable the production of self-expanding stents [27] and grafts [28], bone scaffolds [29], self-tightening sutures [30], drug delivery systems [3], neural interfaces [31], and embolisation devices [32]. In soft robotics, they are used in active elements such as magnetically activated manipulators with variable stiffness [33]; surfaces with controlled roughness [34]; actuators [35], including artificial muscles [36]; and bio-integrated robotic systems [37,38]. In the aerospace industry, SMPs are used in deployable components and thermosensitive elements [39], as evidenced by NASA patent developments [40,41,42]. However, despite these advances, the application of SMPs is limited by their low thermal conductivity, modest mechanical strength, slow recovery rate, and dependence on temperature activation [43,44]. Shape-memory polymer composites (SMPCs), reinforced with nanofillers (carbon-based, metallic, magnetic, or hybrid) [45,46,47] and fibres [48], address some of these issues by enhancing mechanical robustness as well as thermal and electrical conductivity whilst enabling remote activation.

To address the inherent constraints associated with SMPs, particular attention must be paid to the deliberate regulation of their functional performance, most notably the activation temperature (*T*_trans_). A variety of material-based strategies have been proposed to tailor *T*_trans_, such as polymer blending, plasticisation using biocompatible additives, copolymerisation with segments of varying chain flexibility, controlled cross-linking to modulate network density, and the incorporation of functional nanofillers. Each of these approaches provides a versatile pathway for adjusting both the thermal response and mechanical behaviour of SMP systems. Although these strategies effectively mitigate fundamental material limitations, their significance can only be fully appreciated when viewed within the broader framework of contemporary SMP research.

Against this background, it is necessary to position the present study within the current state of the art in SMP research. In recent years, several comprehensive review articles have considerably expanded the understanding of SMP material design principles, activation mechanisms, and emerging application areas. Luo et al. present a comprehensive overview of multifunctional SMPs and their potential applications, with an emphasis on material concepts and translational opportunities, while biomedical-specific considerations such as device performance, degradation behaviour, and regulatory aspects are addressed more selectively [18]. Mirasadi et al. provide a detailed discussion of magnetically responsive SMPs and 4D printing approaches, concentrating primarily on material selection and processing methodologies, whereas biomedical applications and non-magnetic activation pathways receive comparatively limited attention [49]. Sanaka et al. provide a review of stimuli-responsive SMP composites, with a particular focus on thermo-responsive systems. They present a general perspective on composite architectures and blending strategies, with comparatively limited discussion of long-term durability and alternative activation mechanisms [50]. You et al. examine SMPs for biomedical applications across macro- and micro-scales, highlighting a wide range of stimuli and shape-memory behaviours mainly through representative examples, while quantitative performance metrics and long-term biological considerations are treated at a more general level [51]. Fang-Fang et al. focus on the rational molecular design of stimuli-responsive SMPs and SMPCs, providing detailed insights into structure–property relationships from a materials science perspective [52].

Building upon these complementary studies, the present review focuses on approaches to optimise the properties of SMPs by regulating their activation temperature and related material design strategies. The review emphasises a systematic analysis of mechanical performance, biodegradation behaviour, biocompatibility, and functional stability under application-relevant operating conditions. This application-oriented framework guides the rational development of SMP systems for biomedical and soft robotic applications.

### 1.1. A Brief History

The SME was first documented in 1941 by L.B. Vernon, who observed ‘elastic memory’ in poly(methyl methacrylate) (PMMA) resin for dental applications [53]. A pivotal advancement came in the 1960s with A. Charlesby’s demonstration of the SME in γ-irradiated polyethylene (PE), which marked the start of systematic SMP research [54]. At the same time, P. Flory and T. Fox made key contributions to polymer physics, providing insights into network elasticity and thermal transitions that were later crucial for understanding the SME.

Commercialisation began in the 1960s with Raychem’s heat-shrinkable tubes for insulation and packaging films [55], and SMPs were subsequently adopted by the aerospace industry for deployable satellite structures. The 1980s–1990s introduced a new generation of SMPs, including polyolefins, copolymers, and polyurethanes (PUs), with companies such as Nippon Zeon, Kuraray, and Mitsubishi Heavy Industries bringing products to market for automotive and industrial uses [56]. Since the 2000s, SMPs have been increasingly applied in biomedicine, enabling minimally invasive implants such as the IMPEDE-FX RapidFill embolisation device from Shape Memory Medical Inc., which expands in blood vessels to induce thrombosis [57].

The present period of SMP development is defined by a shift toward multifunctional SMPCs and integration into advanced platforms such as 4D printing. Leveraging macromolecular engineering and nanotechnology, modern SMPs exhibit multi-stimuli responsiveness and are being explored for use in soft robotics, smart textiles, wearable electronics, and adaptive biomedical systems, with ongoing research focused on bioinspired and hierarchically structured materials [58,59].

### 1.2. Definitions and Mechanisms

**Figure 1:**
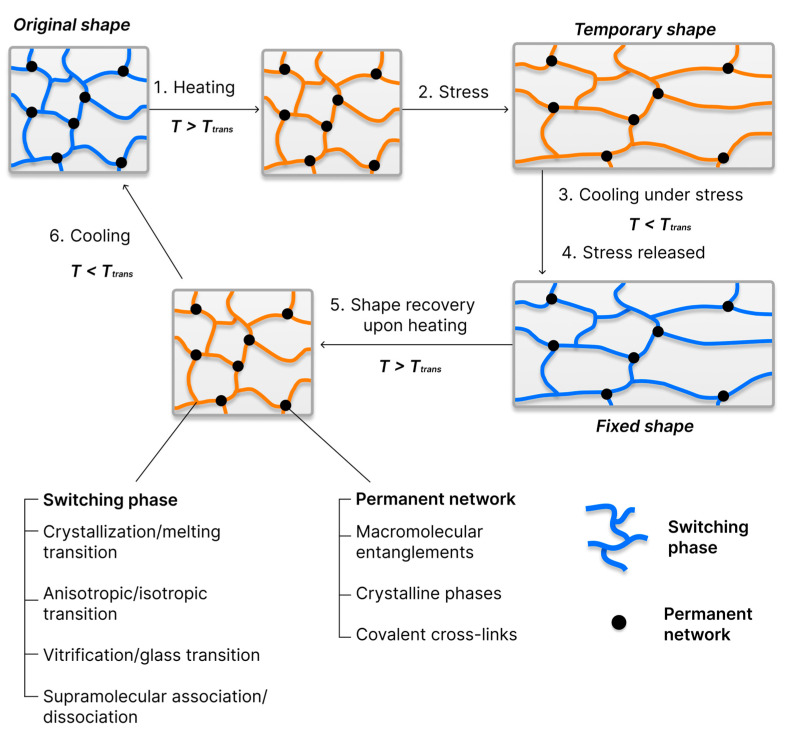
Mechanism of the thermally induced SME. The cycle illustrates how a temporary shape is programmed and later recovered through a thermal transition. The effect relies on a reversible switching phase for shape fixation and a permanent network for shape recovery.

SMPs are a unique class of smart materials that can return to their original shape when exposed to external stimuli. This effect is due to the energy stored during shape programming and the mobility of molecular segments. The recovery rate accelerates with increasing temperature, as segmental rearrangements become easier and relaxation processes proceed more rapidly. SMPs are typically programmed by heating the SMP above *T*_trans_, deforming it into a temporary shape, and then cooling it below *T*_trans_ to fix this shape. Upon exposure to an external stimulus exceeding *T*_trans_, the material tends to return to its original shape, as schematically illustrated in Figure 1.

To exhibit an SME, the polymer must contain an elastic network structure capable of storing and releasing elastic energy. Such a network consists of permanent network points (covalent cross-links or rigid domains) combined with reversible molecular switches, which define the conditions and mechanisms of the shape recovery. The efficiency of the SME depends on multiple factors, including molecular architecture, conformational entropy, morphology, synthesis routes, and programming parameters (e.g., applied strain or stress level, programming temperature, deformation rate, cooling or fixation conditions, and the number of thermomechanical cycles) [55,60,61].

Molecular switches are structural components of the polymer network that undergo reversible transitions in chain mobility or phase in response to external stimuli, thereby enabling both fixation of a temporary shape and subsequent recovery of the permanent shape. Molecular switches are typically regions of the SMP that exhibit a *T*_trans_, which may correspond to a glass transition (*T*_g_), melting transition (*T*_m_), or nematic–isotropic transition (*T*_NI_). When amorphous SMPs are heated above *T*_g_, the polymer transitions from a rigid glassy state to a highly elastic state. In this state, segments gain mobility, enabling deformation through chain orientation and displacement of network nodes. Upon cooling, chain mobility decreases, and the deformation is locked into place. Reheating or applying another stimulus releases the stored entropic elasticity, restoring the permanent shape [57].

Key factors that define SME performance include segment length and flexibility, phase segregation, stereoregularity, the degree of crystallinity, and the type and density of cross-links [62,63,64,65]. For instance, higher crystallinity improves fixation but slows recovery, while amorphous segments allow fast recovery at the expense of stability. Consequently, while the stored elastic energy and recovery stress increase, the overall recovery kinetics are reduced. This is because these rigid crystalline regions impede the motion necessary for a rapid return to the permanent shape. In contrast, SMP systems that are largely amorphous exhibit nearly optimal shape recovery [66]. Additionally, cross-linking density often governs shape recovery force and energy storage capacity more strongly than crystallinity itself [67].

Molecular switches are divided into physical and chemical types. Physical switches rely on reversible intermolecular interactions such as phase transitions or non-covalent bonding (e.g., hydrogen bonds, supramolecular interactions). These processes ensure reversible deformation without altering chemical composition. In contrast, chemical switches rely on dynamic covalent bonds activated by temperature, light, pH, or redox conditions, which can break and reform under stimuli. Such covalent adaptive networks (CANs) provide not only an SME but also self-healing capabilities. Systems that have been thoroughly researched include photoresponsive cinnamate groups undergoing reversible [2 + 2] cycloaddition under UV/visible light [68], thermally labile acylhydrazones/hydrazine [69], disulphide [70], imine [71], isocyanate [72], oxime [73], and boronic/boronate ester bonds [74]. These systems offer multiple reversibility and the capacity to programme SMP for several cycles, including self-healing.

Regardless of the type of switch, netpoints play a central role in maintaining the permanent shape and preventing irreversible creep or slippage of polymer chains. These may be covalent cross-links, physical cross-links (e.g., crystalline domains), or topological entanglements. An SME based on topological entanglement was reported for hard-block-free multiblock thermoplastic polyurethanes (TPUs) consisting of poly(ε-caprolactone) (PCL) and polyethylene glycol (PEG). In these materials, the entanglements slow stress relaxation above the *T*_m_ of the soft blocks long enough to allow elastic deformation and easy shape fixation [75].

In summary, the interplay between molecular switches and netpoints defines the thermal, mechanical, and functional behaviour of SMPs. By tuning these elements, materials can be engineered with tailored *T_t_*_rans_, cycle stability, multi-stimuli responsiveness, and integration potential for advanced biomedical and soft robotic applications.

## 2. Classification of SMPs

SMPs represent a diverse class of smart materials that differ in their chemical composition, internal architecture, activation mechanisms, and functional applications. Previous studies have proposed several classification approaches [76,77].

The most informative criteria from both engineering and chemical perspectives include:

- Type of SME: one-way SME, two-way SME, triple-SME, multi-SME, and multifunctional SME;
- Type of external stimulus: thermal, chemical (redox, pH, specific ions, and chemical agents), solvent, magnetic field, electric field, light;
- Type of polymer network cross-linking: physically cross-linked, chemically cross-linked;
- Chemical composition and molecular design: composites, polymer blends, supramolecular networks, hydrogels, chemically cross-linked polymers, and block copolymers.

**Figure 2:**
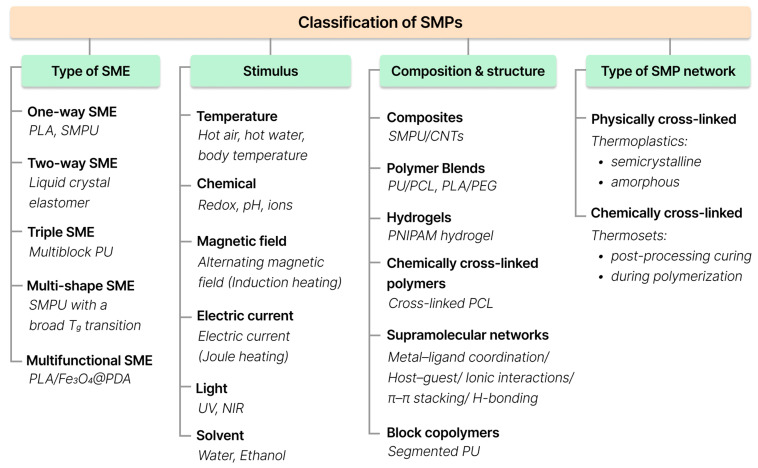
Classification of SMPs based on SME type, stimulus, composition, and network structure.

An overview of these classification criteria is presented in Figure 2, where SMPs are grouped according to SME type, activation stimulus, composition and structure, and network type.

**Figure 3:**
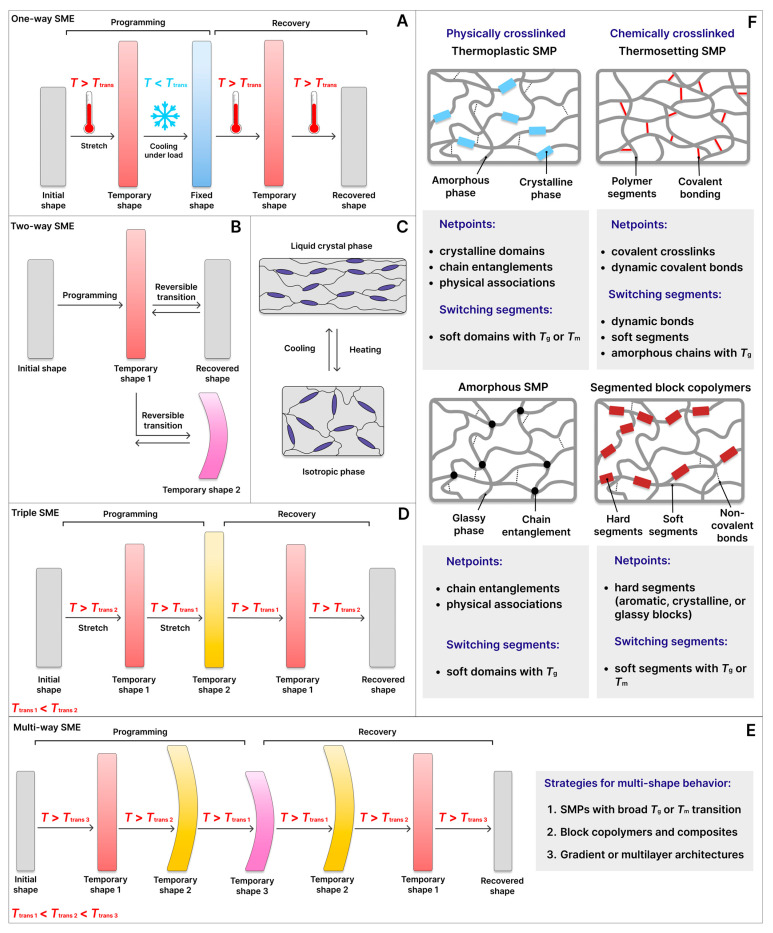
Schematic representation of different SME mechanisms and polymer structural types. (**A**) One-way SME; (**B**) two-way SME; (**C**) two-way mechanism exemplified by a liquid crystal system; (**D**) triple-SME; (**E**) multi-SME; (**F**) various types of polymer structures, including thermoplastic SMP, thermosetting SMP, amorphous SMP, and segmented block copolymers.

Among these, the most universal and practical classification is based on the internal architecture of the polymer network [53]. This framework distinguishes several key SMP classes, described below. Figure 3F provides a schematic illustration of various polymer networks, highlighting the structural differences between thermoplastic polymers, thermosetting polymers, amorphous polymers, and segmented block copolymers, and serves as a visual guide for the discussion in the following sections.

### 2.1. Thermosetting Polymers

Thermosetting polymers (thermosets) are characterised by a permanent network of covalent bonds formed during their curing process (e.g., polymerisation or vulcanisation). This irreversible cross-linking structure grants them exceptional thermal stability, mechanical strength, and resistance to creep, yet it precludes the possibility of melting or reprocessing. Classic examples of thermosets are epoxy [78], phenol–formaldehyde resin [79], and thermoset PU resins [80], as well as cross-linked acrylates [81], polyimides (PIs) [82], and bismaleimides [83]. In these systems, the SME is governed by reversible thermal transitions. Below *T*_g_, the polymer chain segments are kinetically frozen, fixing the temporary shape. Upon heating above *T*_g_, the SMP transitions into a rubbery elastic state, in which the covalent network drives the entropic recovery of the permanent shape defined during synthesis. Recent advances include the development of CANs, which integrate reversible chemical reactions. CANs enable dynamic bond exchange, making it possible to reprogram thermosets’ permanent shape after fabrication [84].

Thermosets’ properties can be tuned by adjusting monomer type and stoichiometry [85,86], chemical functionalisation [78,87], fillers [88], curing conditions (temperature, catalysts, time) [85], cross-link nature [89], and density [90]. Higher cross-link density enhances stability but may reduce recovery efficiency [91].

### 2.2. Thermoplastic Polymers

Thermoplastic polymers, or thermoplastics, are stabilised by physical cross-links such as crystalline domains, glassy segments, hydrogen bonds, or strong intermolecular forces. Unlike thermosets, they lack permanent covalent bonds, which allows repeated moulding, reshaping, and reprogramming without significant degradation. The SME in thermoplastics arises from reversible transitions between glassy and elastic or molten states. Representative materials include TPU, polylactic acid (PLA), PCL, PE, poly(vinyl butyral), and poly(ether–urethane) copolymers. Thermoplastics offer several advantageous properties, including ease of manufacture and moulding, along with the potential to fine-tune properties by utilising plasticisers [92,93], fillers [94,95,96], or chemical modifications [97].

### 2.3. Amorphous Polymers

Amorphous polymers lack long-range order in molecular packing, which imparts isotropy, optical transparency, and high impact resistance. Their SME relies solely on *T*_g_, without crystalline phase contributions. Certain amorphous SMPs display triple-SME behaviour, enabled by broad or heterogeneous *T*_g_ distributions [91]. Compared to semicrystalline SMPs, they offer higher flexibility and easier processing but are more prone to creep and relaxation effects. Temporary shape fixation is achieved through physical entanglements and topological constraints, though recovery is generally slower and less distinct than in crystalline systems. Examples include amorphous TPUs, PMMA, poly(vinyl acetate), and highly amorphous polyesters such as random copolymer poly(D,L-lactide) (PDLLA), which contains a racemic mixture of both D- and L-monomers.

### 2.4. Segmented Block Copolymers

Segmented block copolymers are thermoplastic elastomers composed of alternating soft and hard segments. In these systems, flexible soft segments (e.g., PCL, PEG, oligomers) impart elasticity and high chain mobility, serving as the thermally responsive switching phase. In contrast, hard segments (e.g., urethanes, aromatic polyesters) form rigid domains with high *T*_trans_ that act as physical cross-links, providing structural integrity and defining the permanent shape. The SME arises from the difference in thermal transitions between these phases, whereby the soft segment domain transition governs the switching behaviour, and the hard segment domains maintain the permanent shape. The rigid urethane blocks of shape-memory polyurethanes (SMPUs) crystallise to provide stable fixation, whereas flexible oligomeric chains enable reversible deformation and recovery. SMPUs’ overall properties are determined by the block length, their relative ratio, the degree of phase separation, and the type of cross-linking [98,99], which together control the balance between strength and elasticity [100].

## 3. Types of SME

The diversity of polymer architectures and switching mechanisms leads to a wide range of SME behaviours. These differ in terms of the number of temporary shapes that can be fixed, the activation conditions, and the reversibility of the process. SMEs are generally classified into several main types, ranging from fundamental one-way SME to more advanced two-way SME, triple-SME, and multi-SME behaviours. Figure 3A–E presents a schematic overview of these classifications, illustrating the one-way SME (Figure 3A), two-way SME (Figure 3B), liquid crystal-based two-way SME (Figure 3C), triple-SME (Figure 3D), and multi-SME (Figure 3E).

### 3.1. One-Way SME

A one-way SME occurs when a material programmed into a temporary shape restores its permanent form only once upon exposure to an external stimulus, most commonly heat (e.g., hot air or hot water) (Figure 3A). This behaviour is typical of thermosensitive SMPs, including thermosets, thermoplastics, amorphous polymers, and block copolymers. From a thermodynamic perspective, the one-way SME is governed by reversible thermal transitions. During programming, the SMP is deformed above its *T*_trans_, and cooling below this threshold freezes the molecular switches in a non-equilibrium, high-energy state. Subsequent reheating allows the polymer network to relax, releasing stored elastic and entropic energy, which drives recovery to the permanent shape. The SME process must be repeated for each cycle, but its simplicity and robustness underpin numerous practical applications, including packaging, heat-shrinkable devices, and biomedical implants [101,102].

### 3.2. Two-Way SME

More complex behaviour is observed in the two-way SME, which enables reversible shape changes during cyclic heating and cooling without the need for reprogramming (Figure 3B). This behaviour is commonly exhibited by crystalline thermoplastics, cross-linked thermosets containing reversible domains, liquid crystal elastomers (LCEs), and phase-separated block copolymers. The effect relies on reversible phase transitions, typically crystallisation and melting of polymer domains, or ordering–disordering transitions in liquid crystalline regions (Figure 3C). From a thermodynamic perspective, the driving force arises from the energy difference between ordered and disordered states, while the network’s cross-links maintain stability and reversibility. For instance, LCEs lose anisotropic order and contract upon heating to the nematic–isotropic transition (*T*_NI_), whereas cooling restores mesogenic alignment, resulting in elongation along a predefined orientation.

Advances in 4D printing have enabled precise tuning of the two-way SME, such as in biocompatible TPU nanofibres, where crystallisable soft segments of TPU serve as switching domains, with actuation behaviour modulated by fibre orientation, polymer composition, and composite layer thickness [103].

### 3.3. Triple-SME

The triple-SME allows sequential fixation and recovery of two temporary shapes before returning to the permanent form (Figure 3D). Achieving this effect requires at least three thermodynamically distinct phases with well-separated *T*_trans_: a high-temperature phase stabilises the permanent shape, an intermediate phase governs the first temporary shape, and a low-temperature phase controls the second temporary shape. This behaviour is typically realised in segmented block copolymers [104], polymer blends [105,106], bilayer systems [107], and SMPCs [108], where each phase acts as an independent switching domain. Thermodynamically, the enthalpic and entropic contributions of each phase determine the temporary shape fixation and recovery sequence. For instance, trans-polyisoprene/PCL systems cross-linked with benzoyl peroxide exploit separated crystallisation transitions to programme three shapes within the 37–55 °C range, which is particularly advantageous for biomedical applications [109].

### 3.4. Multi-SME

The multi-SME represents the most advanced manifestation of the SME, in which polymers are capable of storing and sequentially restoring several temporary shapes before finally returning to their permanent shape (Figure 3E). The realisation of the multi-shape SME requires the presence of multiple thermodynamically distinct switching phases with well-separated *T*_trans_. Each switching phase is responsible for the fixation and activation of a specific temporary shape, enabling stepwise shape recovery.

Several strategies have been developed to achieve multi-SME behaviour: (i) utilising polymers with broad *T*_g_ or *T*_m_ regions, which allow programming of multiple shapes within one temperature range; (ii) designing block copolymers and composites with sharply phase-separated domains, each serving as an independent switching unit; and (iii) constructing gradient or multilayer architectures, where different layers are activated under specific environmental conditions.

While thermal transitions remain the most common mechanism, multi-SME can also be activated by external stimuli. For example, hydrogels modified with nitrocinnamate groups have demonstrated light-induced shape recovery, where irradiation modulates cross-link density and phase separation kinetics [110].

To describe these complex behaviours, researchers employ multi-branch thermoviscoelastic models [111,112] as well as hybrid frameworks combining phase transition theory with viscoelastic kinetics [113]. Such approaches allow the prediction of SMP response by accounting for non-equilibrium relaxation in different phases, anisotropy, and large deformations, thereby providing a robust theoretical basis for the design of advanced multi-SME materials.

## 4. Activation Stimuli

The most widely studied and utilised mechanism for activating the SME is thermal activation. Thermal activation can be achieved either directly, by heating the material through contact with hot air or hot water, or indirectly, by converting external physical stimuli into heat. The indirect approach typically relies on functional fillers or chemically active groups embedded in the polymer matrix, which generate heat upon exposure to various external fields, including magnetic fields, electric fields, microwave radiation, or light.

**Figure 4:**
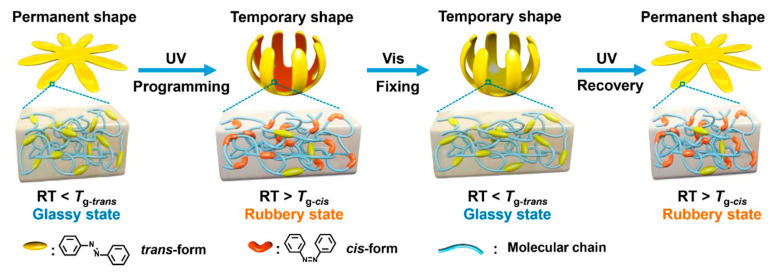
Athermal light-induced SME based on azobenzene isomerisation. Schematic illustration of the photoresponsive shape-memory cycle in an azobenzene-containing polymer network, where reversible trans–cis isomerisation under UV and visible light enables programming, fixation, and recovery of shapes without thermal activation (RT denotes room temperature). Reprinted from Ref. [114] with permission from ACS.

A light-induced SME can be realised through two primary mechanisms: photochemical and photothermal mechanisms. The photochemical mechanism employs light-sensitive molecular switches capable of reversible structural transformations when irradiated at specific wavelengths. Typical processes include trans–cis isomerisation, dimerisation, and [2 + 2] cycloaddition reactions [114,115]. These molecular-level changes induce macroscopic deformation without a significant temperature increase. Common photoresponsive moieties include azo groups, dicarbonates, and other photoreactive covalent dynamic structures. Figure 4 presents a representative example of an athermal, photochemically driven, light-induced SME based on azobenzene isomerisation [114]. This strategy achieves athermal actuation by photoswitching the *T*_g_ of the polymer network. In its permanent shape, the material is in the high-*T*_g-trans_ state. UV irradiation induces trans–cis isomerisation, switching the network into a low-*T*_g-cis_ state, which allows it to be deformed under stress. Subsequent visible-light irradiation drives the reverse cis-trans isomerisation, raising the *T*_g_ back above RT and locking the temporary shape. Finally, re-exposure to UV light again lowers the *T*_g_, releasing the stored entropic energy and enabling shape recovery all at room temperature without thermal input.

Ultraviolet irradiation (*λ* ≈ 365 nm) can convert trans-azo groups to the cis form, fixing a temporary shape, while visible light (*λ* ≈ 450 nm) triggers the breakdown of non-covalent cross-links and restores the original configuration via inclusion complex formation [116]. Key advantages of this mechanism include high spatial and temporal resolution, precise localisation, reversibility, and minimal thermal impact, which reduces the risk of material degradation.

The photothermal mechanism involves the conversion of light energy into heat using embedded photothermal agents. When the local temperature rises above *T*_trans_, it triggers shape recovery. Due to its remote-control capability and high precision, photothermal activation is widely applied in biomedical fields. Effective photothermal agents include carbon nanotubes (CNTs) [117], multi-walled CNTs (MWCNTs) [118], nanocarbon spheres [119], polydopamine (PDA) particles [120], polyaniline [121], graphene derivatives [122,123], and inorganic nanoparticles (e.g., Fe_3_O_4_ [124], urushiol–Fe [125], Au [126]). Au nanoparticles are particularly efficient due to surface plasmon resonance (SPR), enabling intense light absorption and rapid heat generation. For instance, an SMPC containing 0.20 wt.% Au nanorods achieved complete shape recovery within 15 s under laser irradiation (*λ* = 800 nm, *I* = 990 mW/cm^2^) [126]. Reducing the intensity to 130 mW/cm^2^ increased recovery time to 180 s while maintaining high recovery efficiency.

The efficiency of photothermal activation is influenced by wavelength [127], irradiation intensity [128], nanoparticle morphology [129], and concentration [130]. For biomedical applications, near-infrared light (NIR, 700–1100 nm) is preferred due to deep tissue penetration and low absorption. The shape and size of nanoparticles can be used to tune the absorption spectra and heat generation. Au nanostars exhibit a power conversion efficiency (PCE) of 38% at 800 nm, whereas Au nanorods reach 21% under similar conditions [131]. More complex morphologies (nanospikes [132], bellflower [133]) achieve PCE values up to 50–74%. Aspect ratio (AR) adjustments allow precise tuning of plasmon resonance peaks (e.g., AR 2.6 corresponds to 735 nm, AR 3.3 to 820 nm, and AR 5.5 to 934 nm), optimising heating [134]. Nanostars additionally generate stronger local electromagnetic ‘hotspots’ and more hot electrons, enhancing photothermal SME activation [135].

Electrical activation of the SME in polymer composites is typically achieved via Joule heating, in which an applied voltage generates an internal current that is converted into heat, raising the local temperature of the SMP above its *T*_trans_ and thereby triggering shape recovery. This indirect heating mechanism is especially attractive for applications requiring remote or localised actuation without bulky external heaters [136].

Conductive fillers such as CNTs, MWCNTs, reduced graphene oxide (rGO), carbon black, carbon fibres, and metallic or hybrid ferromagnetic particles (Fe_3_O_4_, Fe_2_O_3_, Ni, NdFeB) can be incorporated into an otherwise insulating polymer matrix to form a percolating conductive network [137]. Once the filler concentration exceeds the critical percolation threshold, electrical conductivity increases by several orders of magnitude, enabling efficient Joule heating under relatively low voltages. The morphology, dispersion, and aspect ratio of the fillers strongly influence the onset of percolation and the resulting thermal and electrical response of the composite [44].

For example, SMP composites with embedded three-dimensional graphene foams have demonstrated dramatic improvements in electrical conductivity by 15 orders of magnitude and thermal transport, allowing decimetre-scale samples to recover initial shapes in less than 10 s under simple Joule heating conditions [138]. Similarly, CNT-based SMPCs have exhibited fast electrical triggering at low applied voltages (10 V), with the nanotube network enabling rapid heat generation and uniform temperature rise across the material [139]. These conductive fillers not only facilitate efficient resistive heating but can also enhance mechanical properties, although high filler loadings may compromise processability and shape-memory characteristics if not carefully optimised.

Microwave irradiation is similarly effective, especially with carbon-based fillers that absorb microwave energy, providing fast volumetric heating [140]. For example, graphite-filled 3D-printed SMPCs (10 wt.%) were reported to exceed a *T*_g_ of 67 °C in just 10 s when exposed to 2.45 GHz and 360 W of microwave radiation [6]. In PU/MWCNT composites, microwave exposure successfully triggered the SME and also enhanced mechanical performance, demonstrating that microwave absorption by the carbon fillers plays a key role in volumetric heating and fast actuation [141]. However, microwave activation of the SME also presents notable challenges. Efficient microwave heating critically depends on the type, concentration, and dispersion of microwave-absorbing fillers. SMPCs with low filler content, poor dispersion, or heterogeneous distribution may exhibit non-uniform volumetric heating or incomplete actuation, limiting reliability and performance. Additionally, rapid volumetric heating can generate local overheating or hotspots at high filler concentrations or prolonged exposure, which may cause thermal degradation of the polymer matrix.

Alternating magnetic fields provide an effective method for remote SME activation. Embedding ferromagnetic nanoparticles allows heat generation via magnetic losses (hysteresis, eddy currents), initiating shape recovery without contact. Controlling magnetic field parameters; nanoparticle concentration, orientation, and shape; and sample geometry ensures precise activation [142,143]. Magnetoactive SMPCs combining high-coercivity NdFeB particles with efficiently heated Fe_3_O_4_ particles demonstrate rapid, reversible shape recovery and stable fixation below *T*_g_ [144,145]. Magnetic particle self-organisation into chains under the field further enables targeted modulation of mechanical properties.

Chemical stimuli can also trigger the SME through changes in pH, CO_2,_ humidity, or solvent composition. pH-sensitive SMPs contain ionogenic groups (-NH_2_, -COOH, -SO_3_H) or reversible covalent or non-covalent bonds [54]. For example, β-cyclodextrin-alginate systems fix temporary shapes at pH 11.5 via molecular complex formation, while shape recovery occurs at physiological pH 7.0 with *R*_r_ = 95.7% and *R*_f_ = 94.8% [146]. Similarly, Fe^3+^-alginate/poly(N-isopropylacrylamide) interpenetrating networks undergo rapid shape recovery when exposed to combined pH and solvent changes [12].

Water-sensitive SMPs are extensively studied; for them, water acts as a plasticiser, lowering *T*_g_ by disrupting hydrogen bonds between amide and carbonyl groups [147]. Water penetration increases segmental chain mobility, accelerating shape recovery at ambient or moderately elevated temperatures. Chemical modification, for example, introducing flexible PEG segments into SMPU side chains, enhances moisture sensitivity and mechanical strength in hydrated conditions. For instance, SMPU with a hard backbone and dangling PEG soft segments (SPPU) shows a 2.72-fold increase in Young’s modulus (up to 10.6 MPa) upon hydration, and *R*_r_ = 71.5–100.0% at 37 °C in water, compared with 42.1–100.0% under dry conditions [148].

Similarly, modified poly(vinyl alcohol) (PVA) membranes cross-linked with borax or glutaraldehyde demonstrate how tailored cross-linking can optimise this water-plasticisation effect [149]. This modification enhanced hydrophilicity and drastically accelerated water-induced shape recovery, reducing recovery time from 420 s for pure PVA to 135 s (glutaraldehyde-cross-linked) and 180 s (borax-cross-linked), while achieving complete shape recovery. The mechanism was attributed to a significant drop in storage modulus (from 9711 MPa to 4226 MPa) and *T*_g_ (from 35.03 °C to 12.35 °C) upon water immersion.

The activation stimuli reviewed in this section exhibit clearly differentiated performance envelopes in terms of penetration depth, activation efficiency, and biological safety. Thermal activation is the most universally applicable mechanism, providing high *R*_r_ and stable actuation in SMPs with *T*_g_ or *T*_m_ typically tuned to 30–60 °C. However, heat transfer in biological tissues is limited to millimetre-scale depths and may induce nonspecific thermal damage when temperatures exceed 42 °C. Light-induced activation, predominantly in the near-infrared region, enables localised actuation with response times of seconds to minutes and reported recovery efficiencies above 95% using photothermal fillers such as Au nanorods, graphene, or PDA. Nevertheless, optical penetration in soft tissues is generally restricted to 5–10 mm, and the long-term fate of photothermal nanoparticles remains a concern for implantable devices. Electrical activation of the SME in conductive SMPCs allows rapid actuation (often <10–120 s at 20–30 V) and precise control but requires percolated conductive networks (typically >0.1–1 wt.% carbon-based fillers) and wired power delivery, which limits its clinical applicability. Magnetic activation using alternating magnetic fields offers the greatest penetration depth (centimetre-scale) and fully wireless control; magnetically responsive SMPs incorporating Fe_3_O_4_ nanoparticles achieve near-complete shape recovery under moderate field strengths (30–70 mT), making this approach particularly promising for deep-tissue biomedical and minimally invasive applications, although high filler content can alter mechanical properties, create localised heating, and raise long-term biocompatibility concerns. In contrast, chemical and moisture-responsive activation, including pH- and water-plasticised SMPs, ensures high biocompatibility and activation under physiological conditions but is characterised by slower recovery kinetics and lower spatial controllability. Overall, stimulus selection must reconcile activation depth, safety thresholds, and actuation speed with the intended biomedical application.

To provide a systematic analysis of the various activation stimuli discussed above, Table 1 summarises representative SMP systems together with their mechanical properties, transition temperatures, and shape-memory performance.

In addition to mechanical properties, cyclic durability and fatigue behaviour must also be considered as critical factors governing the long-term reliability of SMPs and SMPCs (e.g., actuators, biomedical implants, deployable aerospace components). Under cyclic thermomechanical loading, SMPs exhibit progressive changes in mechanical response and shape-memory performance due to viscoelastic relaxation, microstructural damage, and irreversible molecular rearrangements.

In amorphous thermosetting SMPs, even in the absence of visible macroscopic damage, cyclic tension leads to degraded stress–strain behaviour and reduced shape-memory response over repeated cycles, primarily due to viscoelastic effects and cumulative hysteresis losses in the polymer network. These effects are often more pronounced at higher deformation amplitudes and rapid cycling conditions [156].

Thermoplastic SMPs typically show moderate cycle resistance. Early studies on polyether-based SMPUs demonstrated small but measurable losses of shape recovery after several hundred cycles, correlating with reduced elastic recovery due to chain slippage and entanglement relaxation. In one example, a commercially available polyether-based SMP showed a 4% decrease in *R*_r_ after 200 bending cycles [99].

In contrast, thermoset SMPs often exhibit superior cyclic stability due to permanent covalent cross-links that suppress viscous flow and irreversible deformation. Thermoset systems tend to retain high shape fixity and shape recovery over repeated cycles, especially when cured architectures are optimised for minimal network relaxation. However, high cross-link density can also reduce toughness and maximum recoverable strain.

A recent study on high-cycle-life SMPs (such as PI-based SMP) demonstrates that molecular features like strong π–π interactions and extensive chain entanglements can preserve both high *R*_f_ and *R*_r_ over more than 1000 cycles, even at elevated temperatures (about 250 °C) [157].

SMPCs introduce additional complexities. Reinforcing fibres (e.g., carbon fabrics) enhance stiffness and load-carrying capacity but also alter cyclic durability. Woven-fabric-reinforced SMPCs maintained high shape recovery and shape fixity over repeated cyclic tests, with failure modes linked to fibre tow bending and matrix damage at high strains [158]. In space and aerospace contexts, SMPCs must survive thermomechanical cycling and environmental stressors (thermal gradients, UV, vacuum), which can induce residual thermal stresses at fibre–matrix interfaces. Thermal cycling often degrades mechanical properties more than shape-memory performance itself, leading to reduced fatigue life in composite laminates despite modest changes in shape recovery behaviour [159].

Studies on different SMP compositions reveal vast differences in fatigue resistance:

– Some UV-curable SMPs exhibit lifetimes of <100 cycles under moderate strain before failure. Others based on tBA–AUD chemistries withstand > 10,000 loading cycles without significant damage, indicating exceptional fatigue resistance [160].
– High-temperature SMPs maintain almost constant *R*_f_ and *R*_r_ over hundreds to thousands of cycles [157]. These results underscore that material chemistry and network design are primary determinants of cyclic durability, sometimes even more than composite reinforcement or processing technique.

For biomedical devices (often single-use), SMPs with moderate cycle resistance are acceptable, whereas soft robotic actuators and deployable aerospace components demand materials and architectures that withstand thousands of cycles with minimal loss of function. Hybrid designs combining tailored network chemistry, reinforced composites, and optimised processing are central to improving fatigue durability in demanding applications.

## 5. Biodegradable SMPs and Biomedical Requirements

Biodegradable polyesters, such as PLA, polyglycolic acid (PGA), and their random or block copolymers with ε-caprolactone and glycolide, are widely used in biomedical applications due to their high biocompatibility and tunable degradation rates. By carefully controlling molecular weight, macromolecular architecture, crystallinity, composition, and morphology, it is possible to precisely modulate mechanical properties, degradation kinetics, and drug release profiles. This versatility makes these materials particularly promising for implantable drug delivery systems and tissue engineering scaffolds.

PLA and lactide-based copolymers (e.g., PLCL) exhibit pronounced SMEs. They are actively studied and applied in medical devices, including stents, vascular grafts, surgical sutures and clamps, controlled drug delivery systems, embolic sponges, orthodontic aligners, and minimally invasive scaffolds [161,162]. A key advantage of PLA-based materials is their processability, allowing fabrication of complex geometries via additive manufacturing, which enables personalised implants.

PLA is a biocompatible, biodegradable aliphatic polyester derived from renewable resources, primarily lactic acid obtained through the fermentation of carbohydrates. It can be synthesised either chemically, through the polycondensation of lactic acid or ring-opening polymerisation of lactide, or biologically, using microorganisms. The polymer’s chemical structure is easily modified during synthesis, enabling variation in macromolecular topology, molecular weight, and stereoregularity. Controlling crystallinity enables the degradation rate of PLA to be adjusted from months to several years.

In addition to PLA-based systems, acrylated poly(glyceryl dodecanoate) (APGD) represents an emerging biodegradable polymer with a notable SME. Unlike conventional thermosensitive SMPs, which require high-temperature programming, APGD undergoes rapid photopolymerisation (UV curing) within seconds. The *T*_m_ can be precisely tuned between 26.7 °C and 36.2 °C by adjusting the acrylation degree and molecular weight [163]. All compositions exhibit a pronounced SME, with deformation fixation at room temperature and full shape recovery at body temperature (37 °C) within one minute. Optimisation of 3D-printed APGD products through thermal post-processing further enhances material performance. A 24 h thermal treatment at 120 °C reduces variability across samples produced by laser cutting, extrusion, and stereolithography (SLA), resulting in *T*_m_ of 30.3–30.7 °C [163].

Beyond thermally activated systems, moisture-responsive SMPs are of growing scientific and practical interest [150]. These include:

- PUs with hydrophilic segments capable of forming reversible hydrogen bonds. Hydration increases polymer mobility by breaking hydrogen bonds, initiating shape recovery.
- Hygroscopic biopolymers, such as PLA and PGA, where water penetrates amorphous regions, plasticising the material and lowering *T*_g_, thus activating the SME under physiological conditions without external heating.
- Hydrogels based on biopolymers (e.g., collagen, gelatine, chitosan, alginates) that respond to humidity, pH, or ionic strength changes.

**Figure 5:**
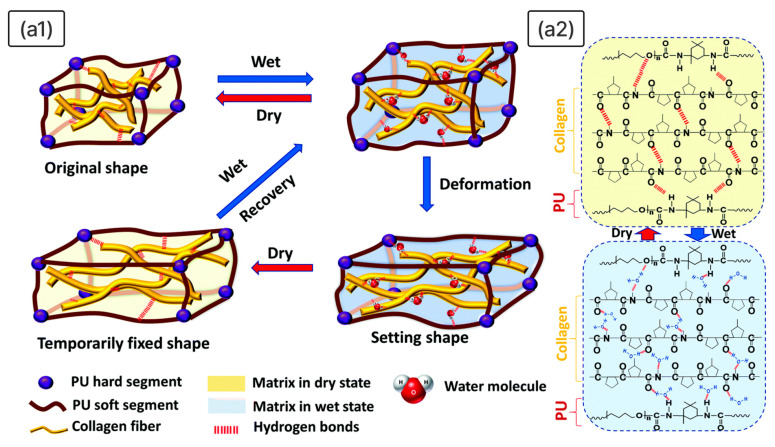
(**a1**) Water-sensitive shape-memory mechanism of SCF/PU biocomposite; (**a2**) behaviours of hydrogen bonds of a dual network during a wet–dry process. Reprinted from Ref. [164] with permission from The Royal Society of Chemistry.

A particularly noteworthy illustration of moisture-sensitive SMP can be found in the skin collagen fibre/polyurethane (SCF/PU) biocomposite, consisting of a PU matrix reinforced with structured collagen fibres [164]. This material mimics the morphology of natural leather and demonstrates *R*_f_ = 99% and *R*_r_ = 90% upon contact with water. The shape-memory mechanism is driven by reversible hydrogen bond disruption and reformation in collagen macromolecules during hydration and dehydration (Figure 5(a1)). In the wet state, water molecules penetrate the SCF/PU, compete with and disrupt the existing hydrogen bonds within and between collagen fibres, as well as at the collagen–PU interface, effectively “unlocking” the fibrous network and allowing deformation (Figure 5(a2)). Upon drying, the water is removed, enabling the reformation of these hydrogen bonds, which lock the temporary shape. Subsequent rehydration reopens the bonds, allowing the elastic recovery force of the PU matrix, aided by swelling pressure and osmotic forces, to drive the material back to its permanent shape.

Beyond its high resilience to repeated deformation cycles, SCF/PU exhibits enhanced biocompatibility compared to pure PU, making it a promising candidate for artificial skin and biomimetic sensors.

### 5.1. Biomedical Requirements

Medical implants are subject to extremely high requirements due to their direct contact with living tissues and long-term, often lifelong, presence in the patient’s body. The key criteria are biocompatibility at the cellular level; absence of carcinogenicity, antigenicity, and mutagenicity; compliance of the material’s mechanical properties with the characteristics of the surrounding tissue to prevent stress shielding and resorption; bioactivity; strength and wear resistance; the presence of a specified 3D porous architecture to ensure osteoconductivity and vascularisation; the possibility of sterilisation without loss of functionality; controlled biodegradation; and compatibility with medical imaging methods. For shape-memory implants, it is also important to ensure a conformable fit to the defect for optimal osseointegration.

The performance of an implant depends on both its composition and structural design, creating a need to balance material characteristics with product architecture. This balance is largely dictated by polymer chemistry and fabrication methods. In all cases, the morphology and mechanical properties of the implant must align with the type of tissue being replaced, particularly with respect to the elastic modulus.

The mechanical properties of implants must be carefully selected depending on the type of tissue. Bone implants require an elastic modulus close to that of cortical bone (3–30 GPa [165]) to avoid the stress-shielding effect, in which excessive implant stiffness leads to a reduction in the load on the surrounding bone and its subsequent resorption. Joint prostheses must withstand cyclic loads without fatigue failure, while soft tissue implants must be flexible and have low elastic modulus values (<1 MPa) [166]. For example, arterial tissue has an elastic modulus of about 0.4–0.8 MPa [167], and nerve tissue has an elastic modulus between 100 Pa and 10 kPa [168].

An important aspect is the sensitivity of mesenchymal stem cells (MSCs) to substrate elasticity, which directly affects their differentiation. Studies have shown that a substrate with an elasticity of 0.1–1 kPa promotes neuronal differentiation of mesenchymal stromal cells, which manifests itself in the filopodia-rich morphology of cells and the expression of neuronal markers [169]; a stiffness of 8–17 kPa, similar to muscle tissue, promotes myogenic differentiation; and a stiffness of 25–40 kPa, characteristic of bone tissue, promotes osteogenic differentiation. These data were confirmed in subsequent studies, which also revealed the influence of substrate stiffness on cell morphology, cytoskeletal organisation, and signal pathway activation [170,171].

The porosity and structure of implants play a critical role in ensuring tissue growth and vascularisation. For bone scaffolds, a system of interconnected pores with a diameter of >300 μm is recommended to ensure new bone and capillary formation [172]. Also, studies show that a pore size of about 100 μm stimulates capillary growth [168], pores of 270 μm are optimal for vascularisation [173], and pores of 300 μm improve metabolism in a developed vascular network, ensuring efficient exchange of substances and oxygen between cells and blood [174]. Micropores (50–100 µm) promote endochondral ossification, while macropores (100–300 µm) improve intramembranous ossification and vascularisation [175].

Porosity must balance mechanical integrity with biological requirements. Bone scaffolds typically have a porosity of 50–90% to allow cell migration and nutrient diffusion while maintaining structural support [176,177]. High porosity (>80%) is necessary for bone and blood vessel ingrowth, as well as for bone oxygenation. However, studies show that even a porosity of 50% combined with a pore size of 200–400 μm and thin walls (~100 μm) provides optimal conditions for blood vessel formation [178].

The surface properties of an implant, such as roughness and chemical functionalisation, significantly impact its biocompatibility and bioactivity. High surface energy improves osteoblast adhesion and promotes bone tissue formation [179]. Among the common surface modifications, PDA should be noted. Modification of PCL scaffolds with PDA leads to a significant increase in cell adhesion and proliferation, as well as enhanced expression of osteogenic markers. PDA-coated scaffolds also demonstrate the ability to form hydroxyapatite (HA) deposits when exposed to simulated biological fluid [180]. PDA-templated nano-HA-reinforced PCL nanofibre scaffolds enhance human MSC adhesion, proliferation, and osteogenesis even without osteogenic additives compared to pure PCL and traditional HA/PCL scaffolds [181]. In addition, mussel-inspired PDA serves as an effective platform for the surface immobilisation of various biomolecules, nanoparticles, and bioactive molecules such as tripeptide arginyl-glycyl-aspartic acid, vascular endothelial growth factor, and bone morphogenetic protein-2 [182,183].

Biodegradable implants are manufactured in various forms depending on the intended application: hydrogels for soft tissue replacement, non-woven materials for skin and hollow organ repair, and porous rigid structures for bone and cartilage replacement. Each design requires an individual approach to ensure the optimal combination of mechanical characteristics, porosity, and surface properties for successful integration with surrounding tissues and performance of its function in the body.

### 5.2. Degradation of SMPs

SMPs designed for biomedical applications must not only exhibit controlled shape-memory behaviour but also undergo predictable and safe degradation in physiological environments. Understanding the degradation behaviour is crucial for ensuring biocompatibility, mechanical integrity during the functional period, and safe resorption of the material. Among biodegradable SMPs, polyesters, such as PLA, are among the most extensively studied due to their well-characterised degradation mechanisms and biocompatibility.

#### 5.2.1. PLA

In the human body, PLA primarily degrades through hydrolysis of its ester bonds [184]. Initially, random chain scission occurs, leading to a gradual decrease in molecular weight. Once molecular weight falls below a critical threshold (typically 10–20 kDa), PLA fragments into soluble monomers and short oligomers that diffuse out of the material and are metabolised by cells [185]. The final hydrolysis product, lactic acid, enters the Krebs cycle and is ultimately metabolised into CO_2_ and H_2_O, which are excreted via the lungs and kidneys, respectively. Cellular processing of low-molecular-weight fragments (<10 kDa) is an important aspect of PLA biodegradation [186]. Macrophages can internalise these fragments via phagocytosis, followed by intracellular degradation with lysosomal enzymes. This immune-mediated clearance is especially relevant for microparticles, fibres, and implants, ensuring safe removal of degradation products and minimising inflammatory responses.

The kinetics of PLA degradation are highly variable, ranging from several months to years, depending on numerous factors: molecular weight and molecular weight distribution, morphology (crystalline, amorphous, stereocomplex), hydrophilicity/hydrophobicity, chemical composition, hydrolysis mechanism (non-catalytic, autocatalytic, enzymatic), the presence and nature of additives (acidic, basic, solvents, medicinal substances), porosity, device size, *T*_g_, and environmental conditions (ionic strength, ion exchange, pH, implantation site) [187,188,189]. In addition, degradation is typically non-linear and can be accelerated by temperature, pH variations, mechanical stress, enzymatic activity, and other physiological factors. In large devices, heterogeneous degradation is often observed, with inner regions degrading faster than surface layers [190]. This behaviour is primarily attributed to autocatalytic bulk degradation, a phenomenon well documented for aliphatic polyesters. During hydrolytic degradation, ester bond cleavage generates acidic degradation products (carboxyl end groups), which accumulate within the interior of thick devices due to limited diffusion. The resulting local decrease in pH accelerates further hydrolysis of polymer chains, thereby creating a positive feedback loop that enhances degradation in the core. In contrast, degradation products near the surface are more readily removed and buffered by the surrounding medium, leading to slower degradation rates at the periphery. This autocatalytic effect results in spatially non-uniform molecular weight loss, pore formation, and mechanical weakening and is therefore a critical consideration in the design of large biodegradable implants with predictable degradation kinetics and structural integrity [184,191,192].

Changes in crystallinity during degradation significantly influence mechanical properties [193]. Semicrystalline PLA degrades more slowly than amorphous PLA because water penetration into crystalline domains is limited [194]. Pistner et al. studied long-term in vivo degradation of poly(L-lactic acid) (PLLA) implants in rats [195]. Semicrystalline microporous samples displayed slower degradation than amorphous injection-moulded samples, highlighting the roles of morphology and crystalline content in controlling hydrolysis rates. For semicrystalline polyesters, degradation begins in amorphous regions and gradually involves crystalline domains. As chains in amorphous regions break and mobility increases, reorganisation into more crystalline structures occurs, resulting in a material with higher hydrolytic resistance than the original polymer.

PLA has been widely studied for biomedical applications, including implants and drug delivery systems. Long-term in vivo studies demonstrate good biocompatibility. Reinforced PLLA plates used in mandibular osteotomy in sheep showed mild foreign body reactions, complete fusion of osteotomies, and almost total resorption after 5 years, with only small PLLA particles remaining [196]. Similarly, 3D-printed PLA-PGA copolymer membranes (lactic–glycolic acid ratio 1:9) demonstrated excellent soft tissue compatibility and formed thin fibrous capsules without hindering hydrolytic resorption [197]. However, PLA degradation may occasionally provoke foreign body responses or inflammation [198]. The severity depends on polymer composition, surface topography [199], morphology [200], stereochemistry [201], animal model [202,203], and implantation site [204].

#### 5.2.2. PCL

PCL degrades very slowly in the human body, typically over a period of 2 years, which limits its use in bone tissue replacement applications [205]. To accelerate degradation, SMP matrices with semi-interpenetrating network (semi-IPN) structures have been developed, incorporating thermoplastic PLLA (*M*ₙ ~ 15 kg/mol) into PCL-diacrylate (PCL-DA) at a weight ratio of 75:25 of PCL-DA:PLLA. These PCL:PLLA matrices retain the excellent shape-memory properties of PCL while demonstrating significantly higher degradation rates in vitro, attributed to phase separation effects. Additionally, the elastic modulus of SMP increases (*E* ~ 23 MPa), which is advantageous for supporting bone defect healing under load.

The primary degradation mechanism of PCL is hydrolysis of ester bonds. Compared to PLA, PCL is more hydrophobic and has a low *T*_g_ of −60 °C, which increases chain mobility but slows water penetration. Despite this, in PCL-based materials, the *T*_m_ of the crystalline domains rather than the extremely low *T*_g_ controls the shape-memory effect, because chain mobility is already high at room temperature, and *T*_g_-based switching would not provide a defined, reversible transition for actuation. This mechanism has been widely reported in studies of PCL-based SMPs for biomedical applications, where tuning of *T*_m_ is essential to ensure thermally triggered actuation at biologically safe temperatures for self-fitting scaffolds [206].

Under phosphate-buffered saline (PBS) incubation at 37 °C, PCL degradation is slow, with approximately 4% mass loss over 6 weeks and a slight decrease in molecular weight during the initial period [207]. Hydrolysis initiates in amorphous regions where water access is easier, and over time, an autocatalytic effect contributes to further chain scission [208]. After implantation, PCL fragments can be phagocytosed by macrophages and giant foreign body cells, followed by intracellular degradation into low-molecular-weight compounds that are subsequently eliminated. Animal studies confirm that PCL does not accumulate in tissues and is typically fully resorbed within months [205]. Degradation rates are influenced by implant size and geometry [209]. Higher surface area accelerates hydrolysis due to greater water accessibility. Incorporation of hydrophilic monomers (e.g., PCL-PEG or PCL-PLA copolymers) further enhances degradation by increasing water uptake [210].

#### 5.2.3. PGD

PGD is a biodegradable thermosetting polymer with a *T*_g_ of approximately 32 °C. At room temperature, PGD behaves as a rigid elastoplastic material, whereas at body temperature, it exhibits compliant, non-linear elastic behaviour. In vitro, 50% mass loss occurs over approximately 16 months in PBS at 37 °C [211]. In vivo studies using subcutaneous mouse models report 25–50% mass loss over 2 months [212]. The degradation rate in vitro depends on cross-link density, while in vivo, enzymatic activity predominates, making this dependence less pronounced. PGD degradation products with varying hydroxyl-to-carboxyl molar ratios (MR_H/C_) affect pH, which influences enzymatic activity and degradation rate [213]. In vivo PGD undergoes surface erosion with mass loss that is linearly proportional to time. Adjusting MR_H/C_ from 2.00 to 0.75 allows linear extrapolation of degradation duration from 9 to 18 weeks, providing a tunable degradation window for implants.

A comprehensive analysis of PGD structure, tissue coverage, endothelialisation, and inflammatory response during transcutaneous implantation of PGD patches (20 × 9 × 0.5 mm^3^) into the pulmonary artery branches of Yorkshire pigs for 3 months is reported [214]. After 3 months in vivo, 5/8 samples demonstrated complete tissue coverage, 2/8 samples demonstrated 85–95% coverage, and 1/8 samples demonstrated limited (<20%) coverage with mild to moderate inflammation. The explanted PGD samples showed a 60–70% loss in volume and a 25–30% loss in mass, as well as a reduction in polymer cross-links. The luminal and mural surfaces, as well as the explant cross-sections, demonstrated signs of degradation.

#### 5.2.4. PU

PU synthesised from biocompatible polyesters, particularly PCL, is biodegradable but typically degrades very slowly. Enzymatic degradation tests in Tris-HCl (0.05 M, pH 8.6) at 37 °C have shown that PLA films degrade completely within 5 days, whereas TPU films lose less than 1% of their mass. Degradation can be accelerated by chemical modification. Introducing oxidatively labile ether groups and hydrolytically labile ester groups (e.g., NTA-DEG) increases the degradation rate [151]. SMPU foams with such modifications show mass loss within 30–60 days in vitro, and the degradation rate can be tuned by varying nitrilotriacetic acid–diethylene glycol (NTA-DEG) content. Incorporation of biodegradable chain extenders, such as oligoglycolic acid (OligoGA), enables precise control of mechanical properties and degradability [215]. PCL-based PUs synthesised with OligoGA and 1, 4-butanediol as chain extenders exhibit gradually increased enzymatic degradation rates with higher OligoGA content. OligoGA segments limit PCL chain mobility, reduce *T*_m_ (~37 °C), and enable shape-memory activation at body temperature.

## 6. High-Temperature SMPs

High-temperature SMPs (HT SMPs) represent a class of functional materials of significant scientific and practical interest due to their unique combination of high thermal stability, mechanical strength, and the ability to undergo reversible conformational transitions under extreme temperatures. Unlike the biodegradable SMPs discussed in Section 5, which are primarily designed for biomedical applications and exhibit shape-memory behaviour near body temperature, HT-SMPs are engineered for demanding environments such as aerospace structures, high-temperature sensors, and critical automotive components [153]. HT SMPs designed for space applications face particularly stringent requirements. They must withstand the combined effects of cyclic thermal loads, exposure to atomic oxygen, ultraviolet and ionising radiation, high vacuum conditions, and the abrasive impact of micrometeoroids and space debris. Consequently, research on HT SMPs prioritises the selection of thermally stable polymer matrices and thermo-resistant modifying components capable of maintaining SME stability at operating temperatures exceeding 200–300 °C.

The most promising heat-resistant SMPs include PI [155], polyamide [216], poly(amide-imide) [217], cyanate ester [152], poly(ether-ketone) [153], polybenzoxazine [218], poly(decamethylene terephthalamide) [219], and various epoxy resins. The development of these polymers has been facilitated by advanced polymerisation techniques, such as polycyclic condensation, polycyclic trimerisation, low-temperature polycondensation, and other modern synthetic approaches [152,220].

Systematic enhancement of HT SMP performance is achieved through targeted molecular design strategies: (i) incorporation of cyclic or aromatic fragments [82,153]; (ii) chemical cross-linking [154]; and (iii) addition of nanofillers, such as CNTs, graphene derivatives, nanoclays, glass fibres, and aluminium nitride particles [221,222]. For instance, the integration of silane-modified aluminium nitride nanoparticles (M-AlN) into thermosetting shape-memory PI matrices enabled the preparation of materials with a *T*_g_ up to 426 °C and thermal conductivity of 5.99 W/(m·K), attributed to the formation of micro-nano hierarchical heat-conducting networks from AlN and Al_2_O_3_ particles [221].

**Figure 6:**
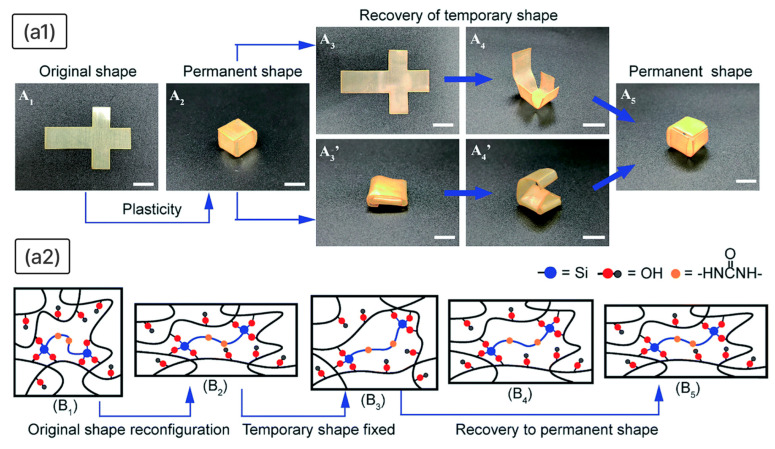
(**a1**) Visual demonstration and (**a2**) mechanism of thermadapt shape memory behaviours of EPSi-0.5 (scale bar: 10 mm). Reprinted from Ref. [154] with permission from The Royal Society of Chemistry.

The introduction of dynamic covalent bonds into polymer networks enables not only high thermal stability and mechanical strength but also reconfigurable shape-memory behaviour. Materials based on dynamic covalent bonds allow for the reprogramming of their permanent shape through network rearrangement. For instance, in epoxy systems cross-linked with a silane agent (EPSi), thermadapt behaviour is enabled by reversible silyl ether linkages (-O-Si-) [154]. As illustrated in Figure 6, the material can be reconfigured via solid plasticity: under heating and stress, silyl ether bonds undergo dynamic exchange, allowing the network to adopt a new permanent shape. The original shape (A_1_) is deformed under external force, and after holding at an elevated temperature (200 °C), the network reorganises into a new permanent form (A_2_) (Figure 6(a1)). This reshaped material can then undergo conventional shape-memory cycles, being fixed into a temporary shape (A_3_ and A_3_’) and recovering to its new permanent 3D structure (A_5_) upon reheating.

The underlying mechanism involves two distinct stages, as shown in Figure 6(a2). First, during network reconfiguration (from B_1_ to B_2_), heating above *T*_g_ under stress allows the dynamic exchange of silyl ether bonds. This bond exchange facilitates the topological rearrangement of the cross-linked network, enabling the material to adopt and fix a new permanent shape through solid plasticity. Once the new permanent shape is set (B_2_), the material enters the second, conventional shape-memory stage (from B_2_ to B_5_). The molecular segments (epoxy phase) can be deformed and frozen into a temporary shape (B_3_) below *T*_g_. Upon reheating above *T*_g_, the stored entropic elasticity drives recovery back to the newly programmed permanent shape (from B_4_ to B_5_), without further altering the network topology.

This dual-mechanism behaviour combines the high thermal stability and mechanical strength of aromatic epoxy networks with the reconfigurability of dynamic covalent chemistry, making such systems promising for high-performance deployable structures.

Multifunctional HT SMPs and their composites represent a particularly promising area of research [18]. Current studies focus on integrative approaches to create self-forming and self-healing SMPs, as well as systems with controlled microstructural ordering. These advanced properties are achieved by modifying network topology, incorporating rigid and flexible segments, and introducing organometallic or ionic cross-linkers, such as Cu^2+^ ions [223].

Despite these advancements, HT SMPs face significant challenges [18]. Achieving simultaneous high thermal stability, mechanical strength, and stable SME remains difficult. Raising *T*_g_ to 250–300 °C often reduces deformability and slows the material’s shape-memory response. Addressing this challenge requires a holistic approach, including optimisation of polymer chemical structure, control of phase behaviour, and incorporation of adaptive microstructures to balance thermal, mechanical, and functional properties.

At the same time, the performance of SMPs, whether designed for high-temperature applications or for biomedical purposes, critically depends on the precise control of the thermal transitions that govern shape-memory behaviour. While HT SMPs are engineered to maintain stability and reversible conformational changes at extreme operating conditions, achieving optimal shape-memory behaviour also requires tuning *T*_trans_ to match the intended service environment. This consideration naturally leads to strategies for adjusting *T*_trans_, which form the focus of the following section. By carefully manipulating polymer composition, segmental mobility, and cross-link density, it is possible to lower or raise the activation temperature of SMPs.

## 7. Tuning the Activation Temperature

### 7.1. Decreasing the Activation Temperature

One of the fundamental tasks in the development of SMPs is the precise control of *T*_g_, as this parameter directly determines the operating temperature for SME activation. For many practical applications, particularly in biomedicine, it is critically important to adjust *T*_g_ to the physiological range of 30–37 °C to ensure optimal functionality in the human body.

In recent years, several strategies have been developed to shift the *T*_g_ of SMPs to physiologically relevant values: (i) introduction of flexible segments with low *T*_g_ via copolymerisation [224,225]; (ii) plasticisation using low-molecular-weight compounds [92,93] or nanoparticles [226]; and (iii) compounding with flexible polymers [227].

It is important to note that *T*_g_ strongly depends on the chemical structure of the polymer, including main-chain flexibility and the volume and polarity of side substituents. Increasing chain flexibility generally decreases *T*_g_. Depending on the polymer system, molecular–kinetic factors (chain mobility) or structural factors (free volume fraction) can dominate *T*_g_ regulation.

#### 7.1.1. Plasticisation

A widely used approach to reducing *T*_g_ and enhancing segmental mobility in SMPs is plasticisation. Plasticisers are low-molecular-weight compounds that occupy the interchain space, weakening intermolecular interactions, increasing free volume, and thereby decreasing *T*_g_ while enhancing elasticity. Plasticisers can be incorporated during polymer synthesis or into the finished polymer melt or solution. Key criteria for plasticisers include thermodynamic compatibility with the polymer matrix, low volatility, and minimal diffusion.

Common plasticisers include PEG with different molecular weights [92], citric acid esters, oligomeric lactic acid (OLA), and other compounds (Table 2). The addition of PEG to the PLA system reduces *T*_g_ and improves flexibility, with the effect dependent on PEG molecular weight: lower-molecular-weight PEG reduces *T*_g_ more effectively but is prone to migration and leaching [228]. Similarly, 5–12.5 wt.% polypropylene glycol (PPG, *M*ₙ 425–1000 g/mol) decreases PLA *T*_g_ and increases elongation at break, but higher concentrations risk phase separation [229]. Oligomeric lactate plasticisers (15–20 wt.%, *M*ₙ = 671–957 g/mol) reduce *T*_g_ from 59 °C to 34–39 °C [230], though potential migration and decreased elastic modulus at higher concentrations remain challenges [231]. Another effective strategy is blending PLA with elastic polymers with low *T*_g_, such as PCL [232]. A PLA/PCL blend forms a two-phase morphology: the PCL crystals act as the switching phase, while the PLA matrix acts as the permanent phase, providing structural rigidity. A 70:30 PLA/PCL ratio achieves high shape recovery at lower activation temperatures compared to pure PLA. Adding compatibilisers (e.g., functionalised isocyanate-terminated PCL diol) improves interfacial adhesion and enhances multi-cycle stability. PLA/thermoplastic starch blends with plasticisers also show controlled *T*_g_ reduction and a pronounced thermoactivated SME [233].

Nanocellulose reduces the *T*_g_ of PU from 47 °C to 34 °C via hydrogen bonding with rigid PU segments, decreasing rigid segment content in the soft phase and enhancing mobility of soft domains at lower temperatures. Green plasticisers, such as citrate esters, offer improved compatibility with PLA [234]. Adding 5–20 wt.% lowers *T*_g_ by tens of degrees while increasing elasticity. Epoxidised vegetable oils (such as epoxidised soybean oil and epoxidised linseed oil) also effectively decrease *T*_g_ and *T*_cc_ while increasing elongation at break [235,236]. For instance, SMPU with sesame-oil-based epoxidised plasticisers achieves *R*_r_ = 85% in 15 min at 36 °C under 93 N load [237]. However, introducing plasticisers can negatively affect shape recovery by increasing crystallisation centres, promoting phase separation, and reducing matrix stiffness [93]. Balancing the ratio of crystalline and amorphous phases is essential to maintain optimal SME characteristics.

The plasticising effect of water is noteworthy [238,239]. SMP composites show *T*_g_ reduction upon water absorption, which increases chain mobility. Heating can remove moisture, restoring the original *T*_g_. Free water exerts the greatest influence on *T*_g_, whereas bound water has minimal effect.

#### 7.1.2. Chemical Modification

Chemical strategies for *T*_trans_ regulation are widely employed [240]. These include copolymerisation of SMP with flexible segments such as ε-caprolactone (ε-CL), trimethylene carbonate (TMC), ethylene glycol, polybutylene succinate, glycolic acid, and polyesters, as well as synthesis of PLA-PCL block copolymers. Table 2 summarises representative SMP systems modified by either physical plasticisation or chemical modification, illustrating the resulting changes in *T*_trans_. Chemical modification, such as copolymerisation or incorporation of functional monomers, typically provides more precise and stable control over *T*_trans_, as the network composition is defined at the molecular level and is less sensitive to migration or leaching of low-molecular-weight additives. In contrast, physical plasticisation using small molecules, oligomers, or nanoparticles often allows broader adjustment of *T*_trans_ over a wide range but may result in inferior thermal or mechanical stability over repeated cycles.

To obtain copolymers of PLA, two main approaches are widely used: directed polymerisation to obtain copolymers of a given composition and structure and graft copolymerisation by reactive mixing (usually extrusion) in the melt in the presence of an initiator and/or catalyst.

In some copolymer systems, *T*_g_ depends almost linearly on monomer content, ranging from 55 °C (pure PLA) to −60 °C (pure PCL) [241]. This allows precise prediction of the required ‘soft’ component ratio to reach the target *T*_g_. Sequential introduction of PLA and flexible polyester blocks (such as PLA-b-PCL) forms two-phase morphologies: a rigid PLA phase ensures temporary shape fixation, while soft PCL domains enable low-temperature mobility [242]. Optimised phase separation yields materials with controlled mechanical properties and cyclic stability, often outperforming simple mixtures.

Furthermore, incorporation of compatible copolymer components reduces *T*_g_ relative to homopolymers. Introducing 6 wt.% of PLA grafted with glycidyl methacrylate significantly decreases *T*_m_ and *T*_g_ while enhancing phase compatibility [243]. The resulting system achieves elongation at break of 370% (compared to 5.5% for pure PLA) with *R*_r_ and *R*_f_ above 90%. Finally, *T*_g_ can also be adjusted by controlling cross-link density and initiator concentration. Keneth et al. tuned *T*_g_ between 6 °C and 20 °C by varying monomer composition [244]. Fatak et al. demonstrated *T*_g_ control through initiator density, which directly affects cross-linking and shape-memory behaviour [245].

**Table 2:** Approaches to reducing the *T*_trans_ of SMPs.

SMP	Fabrication Method	Modification	*T*_trans_	Application	Ref.
Plasticisation					
PLA	Melt blending	PEG (5–20 wt.%)	*T*_g_ = 55.2–48.3 °C	Thermoresponsive devices	[92]
PLA	Solution casting	Tributyl citrate (6–18 wt.%)	*T*_g_ = 33.7–54.8 °C	Dentin tubule sealing	[246]
PLA/BaSO_4_	FDM 3D printing	PEG (20 wt.%)	*T*_g_ = 44.0 °C	Radiopaque ventricular septal defect occluder	[247]
PLA/TPU	Injection moulding	PEG (10 wt.%)	*T*_g_ = 46.0–51.7 °C	Thermoresponsive devices	[248]
PLA	Electrospinning	OLA (20 wt.%)	*T*_g_ = 36.0 °C	Thermoresponsive devices	[249]
PLA	FDM 3D printing	PEG (20 wt.%)	*T*_g_ = 49.1–35.7 °C	Bone scaffold	[250]
Tert-butyl acrylate-co-di(ethylene glycol) diacrylate network (tBA-co-DEGDA)	DLP 3D printing	Nano SiO_2_ (5 wt.%)	*T*_g_ = 37.8 °C	Thermoresponsive devices	[251]
PDLLA	Melt extrusion; orientation-programming	Water	*T*_g_ = 45.8 °C	Medical implants	[252]
PLA	FDM 3D printing	PCL (10–60 wt.%)	*T*_g_ = 47.9–45.2 °C	Spinal cage	[253]
PLA	Injection moulding	Trimethyl citrate (10–20 wt.%)	*T*_g_ = 48.1–19.3 °C	Thermoresponsive devices	[254]
SMPU	Mixing solution	Dibutyl adipate	*T*_g_ = 37.0 °C	Artificial blood vessels	[255]
Chemical modification					
Poly(L-lactide-co-ε-caprolactone) (PLCL)	Solution casting	Copolymerisation	*T*_m_ = 38.0 °C	Wireless nerve stimulator	[256]
Star-PCL-tetraacrylate	Emulsion templating	Ring-opening polymerisation	*T*_m_ = 43.0 °C	Self-fitting vaginal stents	[257]
PU	Water-blown foaming	Two-step polymerisation; water plasticisation	*T*_g_ = 53 °C (dry) *T*_g_ = 25 °C (wet)	Haemostatic foams	[258]
PLCL	Compression moulding	Ring-opening polymerisation	*T*_g_ = 37–40 °C	Oesophageal stents	[259]
PCL-b-PPG-b-PCL diacrylate	DLP 3D printing	Ring-opening polymerisation	*T*_m_ = 53.1–50.7 °C	Tracheal stents	[260]
PCL/HA	Gas foaming	In situ polymerisation	*T*_m_ = 43.4–39.6 °C	Bone scaffolds	[261]
Poly(L-lactide-co-trimethlyene carbonate)/calcium sulphate hemihydrate	Solvent/nonsolvent sintering; freeze-drying	Copolymerisation	*T*_g_ = 26.1–42.3 °C	Bone scaffolds	[262]
Poly(L-glutamic acid)-g-PCL- acryloyl chloride-g-poly(ω-pentadecalactone)	Solvent-casting; particulate leaching	Ring-opening polymerisation; UV cross-linking	*T*_m_ = 36–44 °C	Bone scaffolds	[263]
Poly(rac-lactide-co-glycolide)	Electrospinning	Ring-opening polymerisation	*T*_g_ = 49.1–41.7 (dry); *T*_g_ = 37.6–31.2 °C (wet)	Nerve conduits	[264]
PU	Solution casting	Polymerisation	*T*_m_ = 37 °C	Peripheral nerve stimulation and recording	[31]
APGA	3D printing; UV photo-crosslinking	Polymerisation	*T*_m_ = 21.5–46.6 °C	Adaptive biomedical implants	[265]
PCL-PEG-aniline trimer	Solution casting	Two-step polymerisation	*T*_m_ = 34.5–42.5 °C	Wound dressing	[266]

#### 7.1.3. Influence of Other Factors

One of the key parameters influencing the thermosensitive behaviour of SMPs is the polymer’s molecular weight. A decrease in molecular weight reduces chain entanglement, thereby enhancing segmental mobility and lowering *T*_g_ [267]. In a study on the effect of molecular weight on SMPU films, prepolymers with varying number-average molecular weights were synthesised by adjusting the catalyst content and the NCO/OH molar ratio. The results showed that as *M*ₙ increases, both *T*_m_ and *T*_g_ initially decrease, eventually stabilising at around 200,000 g/mol. Simultaneously, in samples with *T*_g_-determined behaviour, the fixing ability decreased with increasing *M*ₙ, whereas the *R*_r_ increased in both *T*_g_- and *T*_m_-controlled systems. Additionally, *T*_trans_ decreased with increasing *M*ₙ, indicating that SME activation can occur at lower temperatures. It was also demonstrated that the molecular weight of prepolymers, along with the composition, type, and content of comonomers, significantly affects the *T*_g_ of amorphous SMPU networks. Thus, the molecular characteristics of the starting components and the macromolecular architecture provide substantial opportunities for precise tuning of the thermal properties and shape-memory behaviour of SMPU systems.

In fibre structures based on PLA/PCL obtained by electrospinning, γ-irradiation was found to reduce *T*_g_ from 39.3 °C to 34.9 °C, which is attributed to partial degradation of macromolecular chains and the consequent increase in segmental mobility [268]. Moreover, processing parameters during extrusion can be optimised to spatially control *T*_g_ and the PU microstructure [269]. Some studies indicate a strongly non-linear relationship but demonstrate that *T*_g_ can be adjusted by up to 17.8% around a nominal value of 45 °C (ranging from 42 °C to 50 °C) at a fixed thread composition [269]. Atomic force microscopy revealed that increasing the extrusion temperature alters the distribution of microstructural domains, causing rigid polymer domains to coalesce with soft domains. This observation confirms that spatial control extends beyond *T*_g_ regulation and influences the microstructure and degree of crystallinity, which are critical for tailoring the performance of SMPs.

Nanoparticles functionalised with flexible polymer chains can generate interfacial zones of enhanced chain mobility, effectively acting as localised plasticisers that increase the free volume and reduce *T*_g_ [270]. Literature highlights that the surface chemistry of nanoparticles can completely reverse *T*_g_ trends for the same filler type, with grafted or compatibilised surfaces (e.g., PEG-grafted silica) inducing a plasticising effect, whereas bare surfaces tend to restrict mobility [271]. Core–shell particles or nanogels with inherently low *T*_g_ further introduce ‘soft spots’ into the network, and at sufficient loadings, these nanophases decrease the composite *T*_g_ while facilitating shape recovery at reduced activation temperatures [272].

### 7.2. Increasing the Activation Temperature

To specifically increase the value of *T*_g_, several strategic approaches have been developed that focus on enhancing chain rigidity, strengthening intermolecular interactions, and precisely controlling the degree of polymer cross-linking.

#### 7.2.1. Introduction of Rigid Segments and Aromatic Monomers

One of the most effective strategies for increasing *T*_g_ is the incorporation of rigid segments that significantly restrict macromolecular mobility. Aromatic monomers, such as bisphenol A, terephthalates, and various aromatic diols and diamides, have proven particularly effective. For instance, systematically replacing aliphatic segments with aromatic ones in SMPU can increase *T*_g_ from 30 °C to values exceeding 80–100 °C, depending on the rigidity and density of aromatic units. Shape-memory PIs synthesised from aromatic monomers exhibit exceptional thermal stability, with *T*_g_ above 200 °C, and maintain shape-memory behaviour under extreme temperature changes ranging from 150 °C to −150 °C for extended periods (up to 200 h) [273]. Besides this, copolymerisation with high *T*_g_ units, such as polystyrene or PMMA, or the inclusion of linear, rigidly packed macrochains, is another effective method to raise *T*_g_. Block copolymers containing rigid poly(vinyl phenol) blocks can achieve a *T*_g_ of about 200 °C [274]. Dense packing of these chains, combined with reduced flexibility, forms a thermally stable phase that ensures the SME at elevated temperatures.

#### 7.2.2. Increase in the Degree of Cross-Linking

Enhancing the degree of chemical cross-linking between polymer chains restricts their mobility and consequently increases *T*_g_. Multifunctional cross-linkers, such as hexamethylene diisocyanate (HDI) or triethylene glycol dimethacrylate (TEGDMA), along with radical or photochemical curing, are commonly used. Studies have shown that increasing network density significantly raises *T*_g_ compared to linear polymers, although excessive cross-linking can induce brittleness and hinder shape recovery [275]. Physical cross-linking, via mechanisms such as crystallisation or chain entanglement, also contributes to higher *T*_g_ and improved thermal resistance. PUs with hydrophilic segments capable of forming hydrogen bonds allow *T*_g_ adjustment while simultaneously improving resistance to repeated deformation cycles [276].

#### 7.2.3. Introduction of Nanofillers

The incorporation of inorganic nanofillers, such as nanoclays, silica, graphene, and modified CNTs [277], can further enhance *T*_g_ by physically restricting chain mobility and improving interfacial interactions. Nanocomposite SMPs often demonstrate significant *T*_g_ increases, particularly when using functionalised fillers that form hydrogen bonds or adhere physically to the polymer matrix. These bonds act as temporary crosslinks, suppressing chain dynamics and raising *T*_g_. However, careful optimisation of filler chemistry, dispersion, and loading is essential to maintain a balance between higher *T*_g_, mechanical reinforcement, and shape recovery.

Systematic studies have shown that adding CNTs to SMP can significantly increase *T*_g_. The introduction of only 0.2 wt.% CNTs into the SMP matrix leads to a 23 °C increase in *T*_g_ due to strong CNT–SMP interactions [139]. Moreover, the functionalisation of CNTs can further enhance this effect. Jiang et al. demonstrate that when CNTs are covalently bonded to the SMP, the composite’s *T*_g_ increases more significantly, highlighting the role of interfacial interactions [277]. However, the effect of CNTs on *T*_g_ is not universal; it depends on the type of polymer, with CNT addition sometimes increasing *T*_g_ and in other cases having little or even a negative effect [278]. Thus, both CNT content and chemical functionalisation are critical factors in tuning the thermal properties of SMPCs.

**Figure 7:**
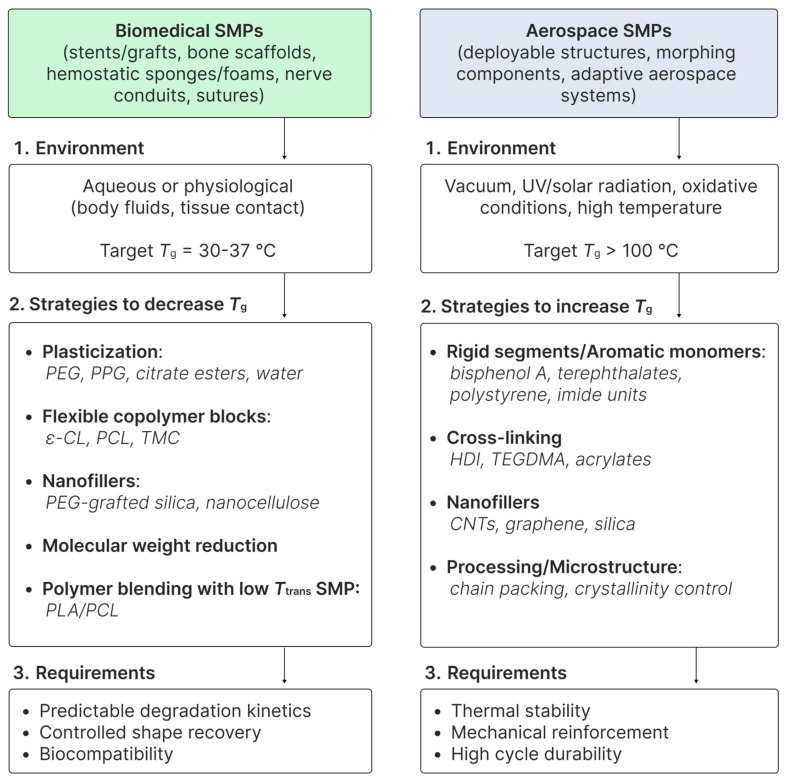
Design strategies for tailoring the *T*_g_ of SMPs for biomedical and aerospace applications.

Overall, the activation temperature of SMPs can be precisely tailored over a wide range through a combination of physical, chemical, and structural design strategies. Approaches aimed at decreasing *T*_g_, such as plasticisation, copolymerisation with flexible segments, molecular weight reduction, and nanoparticle-induced mobility enhancement, are particularly relevant for biomedical applications requiring activation near physiological temperatures. In contrast, increasing *T*_g_ relies on the incorporation of rigid segments, enhanced cross-link density, and reinforcing nanofillers, enabling SMP operation under elevated or extreme thermal conditions typical of aerospace and engineering applications. A schematic overview of these complementary design strategies for tuning the activation temperature of SMPs is presented in Figure 7.

## 8. Fabrication Methods of SMP-Based Materials

The performance of SMPs and SMPCs critically depends on precise control over their morphology, internal structure, and macroscopic shape. Therefore, the choice of fabrication method plays a decisive role in determining the final material properties. Table 3 summarises the main approaches to manufacturing SMP and SMPC materials, their key advantages and limitations, and the suitable materials for each method. This overview provides a clear framework for evaluating the potential of different fabrication techniques and selecting the most appropriate technology based on the desired functional characteristics.

Additive manufacturing offers powerful new routes for SMP fabrication, but each technique has key trade-offs. For example, FFF and FDM are widely used to print thermoplastic SMPs because of their simplicity and low equipment cost [6,279]. However, FFF requires high melt temperatures that can degrade polymer chains, and the layer-by-layer deposition yields anisotropic, rough parts with limited resolution. DIW addresses some of FFF’s shortcomings: it can print complex, support-free shapes from a wider range of materials (thermoplastics, thermosets, and composites). DIW avoids high-temperature melting (preserving material integrity), but it demands precisely formulated inks (shear-thinning and quickly solidifying) and typically suffers from lower print speed. In practice, designers often choose FFF for larger-scale, low-cost parts and resort to DIW when custom ink formulations or multi-material deposition are required.

Vat photopolymerisation methods (SLA, DLP, and related liquid crystal display-based (LCD) resin printing) yield fine-feature SMP components. These techniques cure liquid resins with UV light, giving excellent spatial resolution and surface finish. SLA and DLP can produce highly intricate SMP parts (e.g., microfluidic devices or scaffolds) much faster than extrusion printers. The downside is that only photocurable polymers and often-toxic photoinitiators can be used, and parts may be brittle or limited to specific stimuli. In particular, TPP (direct laser writing) pushes resolution into the nanoscale for microscale SMP actuators, but this requires expensive femtosecond lasers and is very slow. Overall, photopolymerisation offers unmatched accuracy, but at a high cost and with a narrow material choice.

Powder-bed fusion is another additive route. Selective laser sintering (SLS) can quickly fabricate large SMP components without support structures, and it works with strong engineering polymers (e.g., nylon, PEEK, TPU). Its high scan speeds and ability to reuse unsintered powder improve throughput. On the other hand, SLS parts often come out rough and may shrink unevenly; achieving tight dimensional control and smooth surfaces usually requires post-processing [280]. Moreover, the high cost of lasers and polymer powders means SLS is used mainly when its scalability and speed justify the expense. By contrast, traditional injection moulding or casting can mass-produce SMP parts cost-effectively (once the mould is made). These conventional methods yield dense, high-strength SMP components in large volumes, but they lack the design flexibility of 3D printing (e.g., they cannot create internal pores or patient-specific shapes without complex moulds). In short, powder-bed and moulding processes excel at repeatable production of bulk SMP parts, but they sacrifice fine-feature control and often involve expensive tooling.

Beyond 3D printing, fibrous and thin-film techniques enable unique SMP structures. Electrospinning produces nonwoven mats of nano- to micron-scale SMP fibres [281]. These fibrous scaffolds have extremely high surface area and porosity, which are attractive for soft tissue engineering. In practice, electrospinning can generate fibre diameters from tens of nanometres up to a few microns, allowing independent control of mechanical and shape-memory properties via fibre alignment and composition. The trade-off is that electrospun mats are mechanically weak and typically require volatile solvents (raising safety and scalability issues). Melt electrowriting (MEW) addresses some of these issues by extruding molten SMPs under an electric field to write micron-scale fibres in defined patterns. MEW achieves sub-100 µm feature sizes without solvents, giving very precise control over fibre orientation in scaffolds. However, MEW printers are specialised and slow and can currently process only a few thermoplastics and composites. Thus, fibrous SMP methods yield advanced biomedical scaffolds but remain niche due to equipment cost and limited throughput.

Finally, simple casting and foaming remain important for SMPs. Solution casting (and particulate leaching) is a low-cost way to form SMP films or porous foams. For instance, solvent-casting a PU on packed salt particles produced scaffolds with uniform, interconnected pores and high shape recovery [282]. Such methods allow straightforward thickness control and the use of a variety of SMP chemistries. On the other hand, cast films are usually thin (<0.1 mm), slow to produce in large batches, and can suffer from defects (inhomogeneity, cracks) during solvent evaporation. Foaming techniques similarly produce light, highly porous SMP foams (useful for minimally invasive implants), but achieving consistent pore size and distribution is challenging without precise control of the gas-release reaction or templating agents. In summary, casting and foaming are accessible but somewhat crude: they excel at simple geometries and biological scaffolds, yet they offer little control over the microstructure compared to printing or fibre techniques.

In conclusion, no single fabrication method is ideal for all SMP applications. High-resolution methods (SLA, MEW, and TPP) give excellent shape accuracy but are slow and costly, while bulk methods (FFF, SLS) deliver scalability at the expense of surface finish or feature detail. The choice of technique must therefore balance the required resolution, production volume, material compatibility, and end-use. Recent literature emphasises tailoring the fabrication route to the specific SMP chemistry and application. For example, 3D printing can be combined with post-processing or hybrid approaches to optimise both structural complexity and material performance.

**Table 3:** Comparative analysis of SMP fabrication techniques.

Technology	Advantages	Restrictions	Materials	Ref.
FFF (FDM)	High resolution (100–700 µm), availability, printing complex macrostructures	High cost, anisotropy, relatively low resolution (layer thickness > 100 µm), limited compatibility with HT SMP, restricted print size, post-processing, supports required	Thermoplastics (e.g., PLA, PC, TPU, PETG), composites	[6,279]
DIW	High resolution (100–600 µm), support-free printing, wide material selection	High rheological requirements for ink (thixotropic), low printing speed, post-processing	Thermoplastics, thermosets (e.g., methacrylate/acrylate, epoxy-based resins), biopolymers, composites, functional inks	[20,283]
TPP	Ultra-high resolution and accuracy (80–200 nm), no support required	High cost, low printing speed, limited product size, special photoinitiators	Photopolymers (e.g., OrmoComp), thermosets, composites, biopolymers	[284]
SLA	Printing complex 3D structures, high spatial resolution (1–100 µm), structural homogeneity	Limited resin selection, slow print speed, reactive diluents, post-processing, more expensive than FDM	Photopolymers, thermosets, biopolymers	[285]
DLP	High printing speed, high resolution (15–100 µm), accuracy, no support required	High cost, low viscosity (<10 Pa s), reactive diluents	Photopolymers, thermosets	[286,287,288]
SLS	High printing speed, no support required, serial production, minimised waste	High cost, rough surface, post-processing, limited control over shrinkage and deformation	Thermoplastics (e.g., PEEK, PA11, PA12, TPU, PP)	[289,290]
LCD	High printing speed, high resolution (30–50 µm), availability, low cost	Fragility of products, reactive diluents, limited resource of LCD panel, post-processing	Photopolymers, thermosets, composites	[291]
(Direct laser writing) DLW	Complex 3D structures, high resolution (0.1–0.5 µm), no support required, minimal post-processing	SMP microprinting is not worked out, low printing speed, high cost	Photopolymers, typically thermosets, composites	[292]
Electrospinning	Production of ultrathin fibres (from 10 nm to 10 µm) with a large specific surface area, wide range of materials	Difficult to scale, toxic solvents, low mechanical strength	Thermoplastics, thermosets, composites, biopolymers	[293]
MEW	High-resolution, solvent-free, precise control over fibre placement	Limited material options, high cost, low speed, restricted build size	Thermoplastics (e.g., PCL, PLA, PU), composites, biopolymers	[294]
Solution casting	Simple and low-cost method, good film thickness control (0.02–0.10 mm), compatible with various polymers	Thickness is limited, low scalability, possible inhomogeneities and defects, slow process, toxic solvents, poor mechanical properties	Thermoplastics, thermosets, composites	[295]
Injection moulding	High speed, serial production, accuracy, complex geometries possible, wide range of materials	High cost, long setup time, possible defects, not suitable to prepare porous architecture	Thermoplastics, thermosets, composites	[296]
Foaming	A simple and affordable method to prepare light and porous structures with a large surface area, wide choice of materials	Pore size control, non-uniform structure	Thermoplastics, thermosets	[297]
Spin coating	Simple/high speed process, thickness control (from 10 nm to 220 µm), multilayer structures can be formed, wide choice of materials	Requires control of solution viscosity and rotation speed, limited to flat substrates	Thermoplastics, thermosets	[298]

## 9. Biomedical Applications of SMPs

One of the most promising areas of modern biomedical engineering is the use of SMPs to create medical implants and devices for minimally invasive cases [299]. Owing to their ability to be delivered in a temporary compact shape and recover a programmed shape in situ under physiological stimuli, SMP-based systems reduce surgical trauma, decrease access size, and may shorten patient recovery times.

SMPs offer several specific functional advantages in biomedical applications, as well as practical benefits. Their ability to respond to stimuli allows them to adapt to complex anatomical geometries with great precision, improving the conformity between the device and tissues and reducing mechanical mismatch at the tissue–implant interface. SMPs can be designed to have tunable mechanical properties, *T*_trans_, porosity, and degradation rates. This enables them to provide temporary structural support while gradually resorbing, which minimises the need for secondary surgeries and long-term foreign body presence. The ability of SMPs to morph and recover shapes in response to non-invasive stimuli (e.g., moisture, pH) also facilitates on-demand deployment and activation within the body, broadening their utility beyond static implants to dynamic, adaptive devices. In advanced designs, SMPs have been integrated into fibre or composite architectures to enable controlled drug delivery with spatially and temporally regulated release, as well as rapid self-tightening behaviour that is useful for smart sutures and multifunctional wound care devices.

Recent studies demonstrate SMPs’ potential in several clinically relevant areas. Porous SMP-based foams have been designed for rapid haemostasis [300], while osteoconductivity 3D scaffolds fabricated via additive manufacturing techniques show promise for bone regeneration. These structures not only provide mechanical support to damaged tissues but also enhance cell adhesion, proliferation, and differentiation, thereby improving healing processes and promoting integration of the implant with surrounding tissues. Moreover, SMPs can be functionalised with bioactive components, such as natural biopolymers (e.g., PDA [301], collagen [302]), signalling molecules (growth factors), and peptides [303], further enhancing biocompatibility and enabling targeted modulation of cellular responses.

A representative example is SMPU foam developed for the treatment of intracranial aneurysms [304]. In a lateral carotid artery aneurysm model in pigs, the SMP foam was compared to traditional metal coils. After 90 days, aneurysms treated with SMP foam showed accelerated healing compared to the control group. By day 180, the aneurysm cross-sectional area in the experimental group decreased by 89–93%, whereas the control group showed a reduction of only 18–34%. These results demonstrate the high efficacy of SMPU foam in promoting thrombus formation and tissue regeneration without eliciting significant inflammatory responses.

**Figure 8:**
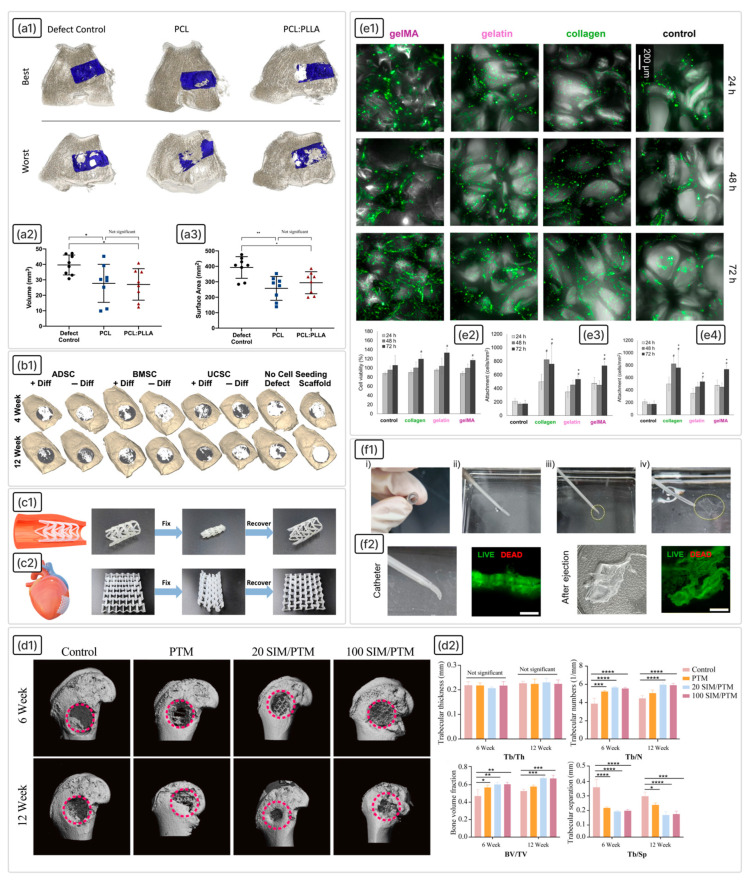
Representative demonstrations of biomedical applications of SMPs in tissue engineering, bone regeneration, vascular devices, and minimally invasive delivery systems. (**a1**) Representative 3D micro-CT reconstructions demonstrating the best (top row) and worst (bottom row) bone healing, as assessed by new bone volume (mm^3^) deposition (blue cylinder) within the defect for each treatment condition (*n* = 8 per treatment group). New bone volume (mm^3^) and surface area (mm^2^) were quantified using 3D reconstructions generated from 3D micro-CT scans of each specimen. Scatter plots of bone volume (**a2**) and bone surface area (**a3**) demonstrate individual values from each specimen. Bars within groups denote the mean and standard deviation. Defect control specimens exhibited significantly greater bone volume and surface area than both SMP compositions (* *p* < 0.05, ** *p* = 0.0054). Reprinted from Ref. [29] with permission from Wiley. (**b1**) 3D reconstructions show the bone regenerated in the defect volume for each group. Reprinted from Ref. [305] from Wiley. 4D-printed PCL-AD-4 components: (**c1**) vascular stent and (**c2**) myocardial patch. Reprinted from Ref. [306] with permission from Science. (**d1**) Micro-CT 3D reconstruction images of defect sites at different time points after scaffold implantation, (**d2**) newly formed bone mass (BV/TV; BV, newly formed bone volume; TV, total volume), trabecular separation (Tb.Sp), thickness of newly formed bone trabeculae (Tb.Th), and number of newly formed bone trabeculae (Tb.N) (* *p* < 0.05, ** *p* < 0.01, *** *p* < 0.001, **** *p* < 0.0001, *n* = 5). Reprinted from Ref. [307] with permission from Elsevier. (**e1**) Merged z-stack brightfield and GFP images of cells attached to PU foams. Changes in cell (**e2**) viability, (**e3**) attachment, and (**e4**) spreading over 24, 48, and 72 h on PU foams (* *p* < 0.05 comparing 72 h time point of bioactive foams to that of the control; # *p* < 0.05 comparing 48 and 72 h time points to the 24 h time point within a sample). Reprinted from Ref. [300] with permission from ACS. (**f1**) The injectable ultrathin porous membrane (IUMP) demonstrates self-expandability when immersed in a 37 °C water bath. (i) Placement of the fixed IUMP, after deformation and freezing, at the catheter hub. (ii) Preparation for injectability evaluation by immersing the catheter in water at 37 °C. (iii) Image of the IUPM passing through the catheter. (iv) The IUPM recovering its original shape in 37 °C water. (**f2**) Images illustrating the catheter injectability of the IUPM. Fluorescence microscopy images showing live/dead staining of ARPE-19 cells 24 h after seeding on IUPMs. Live cells are stained green, and dead cells are stained red (scale bar = 50 mm). Reprinted from Ref. [308] with permission from the Royal Society of Chemistry.

SMPs also show significant promise in bone tissue engineering. In vivo studies of PCL-based SMP matrices in rabbit distal femoral condyle defects [29] demonstrated excellent biocompatibility, support for bone tissue growth, effective fibrous tissue infiltration, and minimal inflammatory response. Two compositions were evaluated: pure PCL and a PCL/PLLA blend. Representative outcomes are shown in Figure 8(a1–a3), where both materials successfully integrated with surrounding bone tissue under mechanical stress. Figure 8(a1) presents 3D micro-computed tomography (micro-CT) reconstructions illustrating the best and worst cases of bone healing for each treatment group. Quantitative analysis (Figure 8(a2,a3)) revealed that while both SMP compositions supported bone formation and successfully integrated with the surrounding bone tissue under mechanical stress, they resulted in statistically significantly lower new bone volume and surface area compared to the untreated defect control. This indicates that the SMP scaffolds provide a compatible environment for osteointegration and tissue infiltration in a load-sharing environment, without inhibiting the body’s natural healing response.

PCL-based SMP matrices modified with PDA were combined with MSCs to regenerate critical-size skull defects in a rat model [305]. After 12 weeks, bone volume in groups with pre-differentiated MSCs increased to 15.69%, compared to 4.41% in controls. Three-dimensional reconstructions show the bone regenerated in the defect volume for each group shown in Figure 8(b1). Mechanical testing confirmed significant improvements in strength and osteointegration, demonstrating the potential of such hybrid systems for bone regeneration.

Recent advances also include the development of biocompatible PU elastomers, such as PCL–adipic dihydrazide PU with an average of four PCL segments per prepolymer (PCL-AD-4), which mimic the mechanical behaviour of native skin [306]. This material exhibits a pronounced SME at body temperature, with an initial elastic modulus of 3.7 MPa that increases 86-fold under strain and a true tensile strength of 1.42 GPa. Its fracture energy (384.7 ± 18.9 kJ/m^2^) surpasses that of native skin by a factor of 107 while maintaining functionality over 100,000 deformation cycles. The SME is activated at physiological temperature (*T*_m_ = 37 °C) by controlled crystallisation of PCL segments, ensuring almost complete shape fixity (*R*_f_ = 100%) and high shape recovery (*R*_r_ > 94%). Comprehensive in vitro and in vivo studies confirmed biocompatibility, with minimal apoptosis (<5%), absence of haemolysis, and no inflammatory response. Transcriptomic analysis revealed activation of genes related to cell proliferation and DNA repair. Figure 8(c1,c2) shows representative 4D-printed PCL-AD-4 constructs, including a vascular stent and a myocardial patch, all of which undergo compression and expansion with excellent shape recovery, maintaining integrity without deformation.

A promising strategy involves poly(l-lactide-co-trimethylene carbonate)/simvastatin/mesoporous bioactive glass (PLTMC/SIM/MBG) composite scaffolds with an SME for bone tissue regeneration [307]. These scaffolds exhibit high porosity (78.5 ± 1.5%) and substantial mechanical strength (66.33 ± 1.44 MPa with 30% MBG). The SME (*T*_g_ = 37.9 °C) allows rapid shape recovery of the original shape within 10 s, enabling precise adaptation to bone defect geometry. MBG and SIM facilitate controlled release of osteogenic ions (Ca^2+^, SiO_4_^4−^) and pharmacological agents, creating an optimal bioactive microenvironment. In vitro experiments demonstrated enhanced osteogenic differentiation of MSCs via Wnt signalling, and in vivo studies in rats showed increased bone volume (BV/TV), improved trabecular thickness (Tb.Th) and number of newly formed trabeculae (Tb.N) (Figure 8(d2)), active angiogenesis, and mineralisation along scaffold pores, with structural integrity maintained over 12 weeks. The progressive bone regeneration within the defect sites over time, as visualised by 3D micro-CT reconstructions (Figure 8(d1)).

Bioactive PU foams with an SME, modified with collagen and gelatine, have been developed to enhance wound healing [300]. These foams reduce blood-clotting time by 30–40% and increase platelet adhesion by 50%. The thermally activated SME (*T*_g_ = 55 °C, dry; *T*_g_ = 37 °C, wet) restores the original porous structure in 20 s at body temperature, ensuring a tight, conformable fit to complex wound surfaces. After modification with bioactive components, NIH/3T3 cell viability exceeded 95% after 24 h, with a 40–60% increase in proliferation after 72 h. Dynamic perfusion tests showed effective redirection of up to 70% of blood flow during coagulation, maintaining low density (<0.1 g/cm^3^) and achieving a dry elastic modulus of 60 kPa after gelatine methacrylate (gelMA) modification. Figure 8(e1) shows uniform cell adhesion and proliferation throughout the foam structure. Quantitative analyses of cell viability, attachment, and spreading over 24, 48, and 72 h are shown in Figure 8(e2–e4). All foams demonstrate excellent cytocompatibility, with cell viability above 95% after 24 h. Over 72 h, the bioactive foams exhibit a statistically significant increase in cell viability compared to the control PU. In contrast to the control samples, where cell attachment diminishes after 48–72 h, indicating limited proliferation, gelatine-, collagen-, and gelMA-modified foams promote greater initial adhesion and continuous cell spreading and growth throughout the 72 h period.

Jo et al. successfully developed an injectable ultrathin porous membrane (IUPM) using a blend of PLCL and PLGA, optimised for retinal tissue engineering [308]. The PLCL1/PLGA1 blend ratio demonstrated shape-memory properties, with an *R*_r_ of 68.67% and an *R*_f_ of 97.06%, enabling self-expansion at 37 °C for minimally invasive catheter delivery. Figure 8(f1,f2) illustrates the catheter injectability and shape-memory behaviour of the IUPM. The membrane can be deformed and loaded into a catheter, and upon immersion in 37 °C water, it self-expands to its original shape, while fluorescence images confirm high ARPE-19 cell viability 24 h after seeding. The membrane exhibited a Young’s modulus of 1.77 MPa and a high modulus of resilience (1.05 × 10^6^ J/m^3^), indicating excellent elasticity and energy absorption. Fabricated via vapour-induced phase separation at 60% relative humidity, the IUPM featured a nanoporous structure (average pore size: 520 ± 350 nm) and an ultrathin thickness of 300 nm, closely mimicking Bruch’s membrane. The scaffold supported robust ARPE-19 cell viability, enhanced epithelial barrier formation with high ZO-1 and F-actin expression, and upregulated key genes (CLAUDIN, CRALBP). Ex vivo experiments confirmed successful subretinal injection and expansion of cell-laden IUPMs, maintaining high cell viability and demonstrating potential for clinical application in treating retinal degenerative diseases like age-related macular degeneration.

Despite significant progress, SMPs face several limitations that stop them from being used widely in clinics. One major limitation of SMP foams is the lack of control over shape recovery kinetics and final expansion volume in vivo. This can result in excessive local pressure, potentially leading to tissue compression or ischaemia in confined anatomical spaces, such as intracranial cavities. While SMP foams are being explored for haemostatic applications, achieving degradation profiles and actuation kinetics that align with tissue healing remains challenging, and current formulations still require optimisation for clinically relevant performance [151].

Additionally, sterilisation using γ-irradiation or ethylene oxide has been shown to shift *T*_g_ and reduce shape recovery performance, which negatively affects shelf stability and reproducibility. Thermal and chemical sterilisation can compromise polymer networks and alter mechanical and thermal behaviour in SMPs and similar polymer scaffolds, making sterilisation a critical processing concern in clinical translation [309].

While SMP scaffolds permit minimally invasive implantation and good defect conformability, their mechanical properties are inadequate for load-bearing applications. The typical elastic modulus of SMP scaffolds ranges from 10 MPa to 500 MPa, which is substantially lower than that of native cortical bone (7–30 GPa), meaning these materials are not inherently suited to bear physiological loads without additional reinforcement or composite design strategies. This mechanical mismatch is recognised as a key challenge for SMP adoption in bone tissue engineering [310].

Several in vivo studies report significantly lower new bone volume and surface area in SMP-treated defects than in untreated controls, indicating limited intrinsic osteoinductivity unless bioactive fillers, growth factors, or cells are incorporated to actively enhance bone regeneration. However, long-term in vivo studies on degradation and remodelling behaviour in SMP scaffolds remain scarce, highlighting the need for more comprehensive evaluation of biological performance [310].

SMP-based vascular stents and patches exhibit excellent compressibility and recovery but generally demonstrate lower radial strength and fatigue resistance than SMA devices. Although specific work on fatigue life for SMP vascular devices is still emerging, polymer-based stents are known to have mechanical limitations such as lower moduli and less robust fatigue behaviour compared with traditional metal counterparts, which can limit long-term vascular performance and durability [311].

Furthermore, shape recovery at physiological temperature often takes tens of seconds to several minutes, which may be insufficient for time-critical deployment and rapid actuation in the clinical setting [312]. Many thermally activated SMP systems rely on passive body heat or fluid activation, but the kinetics are substantially slower than required for minimally invasive procedures.

Additionally, the low radiopacity of polymeric SMP membranes and foams makes in vivo visualisation and positioning difficult, often requiring contrast additives that may affect shape-memory performance. SMP foams have been demonstrated to be almost invisible under X-ray imaging due to very high porosity, requiring the incorporation of heavy atoms such as tungsten to achieve clinically relevant radiopacity [313].

SMP-based medical devices face challenges related to manufacturing scalability, batch-to-batch variability and sensitivity to processing and sterilisation conditions across applications. Furthermore, most studies remain limited to short- or medium-term animal models. This underscores the need for standardised long-term in vivo evaluation prior to clinical translation.

## 10. Application of SMPs in Soft Robotics

A new class of rigid-flexible interwoven polymers (RFIPs) with an SME has been proposed, synthesised by molecular interweaving of PU and PI chains using Cu(I) ions as coordination centres [223]. These materials demonstrate an exceptional combination of mechanical characteristics: tensile strength up to 91.4 MPa, elasticity with elongation at break up to 1185%, and impact strength of 448 MJ/m^3^, which exceeds traditional analogues by 2–2.5 times. The microphase-separated structure, combined with the formation of strong coordination and hydrogen bonds, provides outstanding resistance to cyclic loads. The materials retain their structural integrity after 10,000 tensile cycles and demonstrate complete shape recovery when heated to 80 °C.

**Figure 9:**
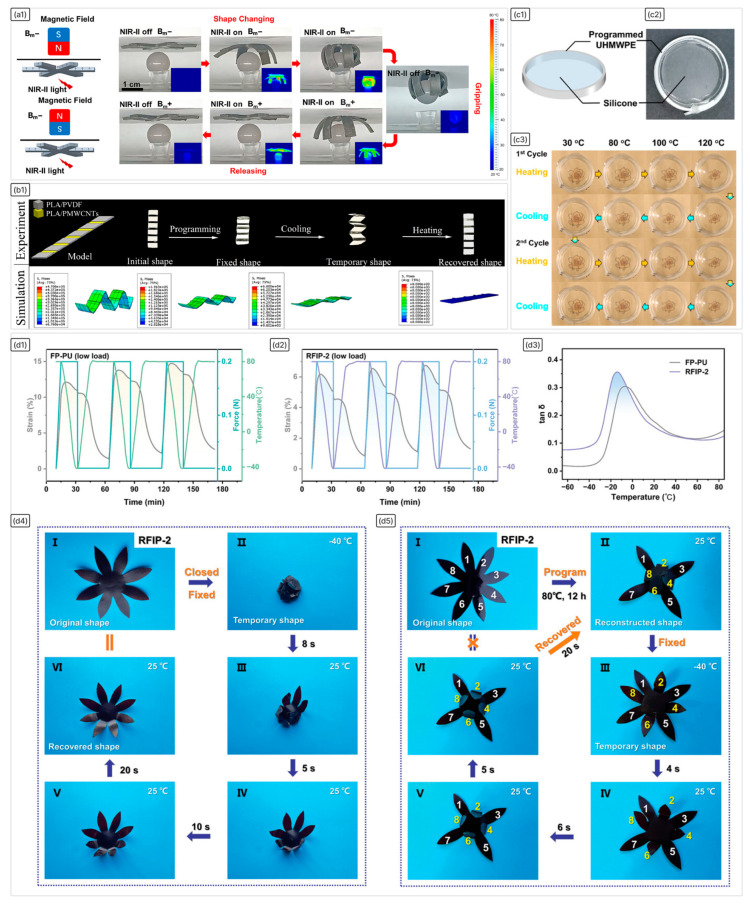
Representative demonstrations of multifunctional SMP systems. (**a1**) Magnetisation of a six-arm magSMP gripper and direction of applied magnetic field, with demonstration of gripping, lifting, and releasing a ball under synergistic NIR-II light (1064 nm, 0.3 mW/cm^2^) and magnetic field (110 mT). Reprinted from Ref. [314] with permission from Elsevier. (**b1**) Simulation and experimental validation of bio-inspired snake-like motion in electroactive SMPs. Reprinted from Ref. [315] with permission from Elsevier. (**c1**,**c2**) Deformable silicone rubber lens with a reversible multiple-shape-memory ultra-high-molecular-weight polyethylene (UHMWPE) ring actuator. (**c3**) Rose images observed through the temperature-tuned lens. Reprinted from Ref. [316] with permission from Elsevier. (**d1**) Shape-memory cycle DMA curves of FP-PU under 0.2 N load. (**d2**) Shape-memory cycle DMA curves of RFIP-2 under 0.2 N load. (**d3**) Tan δ curves of FP-PU and RFIP-2. (**d4**) Photographs showing shape fixing and shape recovery of RFUP-2. (**d5**) Photographs illustrating shape reconstruction and repeated shape-memory process of RFUP-2. Reprinted from Ref. [223] with permission from ACS.

Comprehensive analysis using molecular dynamics and experimental techniques confirmed that the intertwined architecture significantly increases the system’s cohesive energy (768.65 kJ/mol) and contributes to effective energy dissipation through the reversible breaking of dynamic hydrogen and coordination bonds between Cu(I) and ligands. The dynamic mechanical analysis (DMA) curves for the shape-memory cycles of FP-PU and RFIP-2 under a 0.2 N load (Figure 9(d1,d2)) and their comparative tan δ curves (Figure 9(d3)) reveal high shape recovery rates that improve with cycling, reaching up to 96.6%. The one-way SME was visually confirmed through photographs demonstrating the shape fixing and shape recovery process of RFIP-2 (Figure 9(d4)). The material can be deformed into a temporary shape at low temperature and then quickly recover to its original shape at room temperature. Furthermore, the dynamic nature of the bonds enables multiple reprogramming. A series of photographs illustrates this shape recovery and repeated shape-memory process (Figure 9(d5)), where a ‘sunflower’-shaped RFIP-2 sample is programmed to a new permanent shape and subsequently cycles between temporary and programmed shapes. This demonstrates the material’s exceptional capability for precisely controlled, reconfigurable shape changes.

An innovative class of composite magnetic SMPs (magSMPs), prepared using DLP 4D printing technology, is reported [314]. The material integrates a polymer matrix with an SME and highly anisotropic NdFeB particles, providing exceptional photoconversion efficiency (42%) and pronounced magnetic sensitivity. The synthesised structures demonstrate rapid and reversible switching between conformational states under the influence of light in the near-infrared NIR-II range (1064 nm) and precise control of deformations under the combined influence of a magnetic field (up to 50 mT) and optical radiation. Their application in robotics has been demonstrated using biomimetic structures such as soft grippers (Figure 9(a1)), which successfully grasp, lift, and release objects under combined magnetic and optical stimulation. The material retains its stability during repeated reprogramming (up to 10,000 cycles) with *R*_f_ = 98% and *R*_r_ = 92%. This synergistic control overcomes the fundamental limitations of traditional actuators, opening new prospects for remotely controlled adaptive systems.

Significant progress has been made in the development of electroactive SMPs for 4D printing using FDM based on a 50/50 binary mixture of PLA and polyvinylidene fluoride (PVDF) (sample P5F5) [315]. The material demonstrates partial compatibility of components with a decrease in the *T*_g_ of PLA from 69.99 °C to 60.26 °C and balanced mechanical characteristics: elastic modulus 950 MPa, tensile strength 32 MPa, and relative elongation at break 8.5%. The incorporation of 7.5% MWCNTs ensured optimal filler dispersion and high electrical conductivity, which is necessary for effective Joule heating. When a voltage of 10 V and a current of 0.14 A were applied, the PLA/7.5% MWCNT nanocomposite reached an activation temperature of 80.28 °C in 10 s, which is sufficient to overcome the *T*_g_ of the polymer matrix. The optimised P5F5 composition with PLA/7.5% MWCNT joints demonstrated record values for the shape recovery (*R*_r_ = 98.6%) and shape fixation (*R*_f_ = 99.6%), maintaining high performance after five programming cycles. Infrared thermography confirmed the possibility of local heating up to 104.44 °C for selective activation of complex structures. The developed multiphysics model in Abaqus accurately predicted the temperature field and shape recovery kinetics, which made it possible to implement functional devices: bionic structures with opening ‘butterfly wings’, a moving ‘snake’ (Figure 9(b1)), and a soft robotic manipulator with precise independent finger control (*R*_f_ > 99%, *R*_r_ > 98%).

Finally, a fundamentally new concept of reversible SMPs has been proposed for the first time, where an amorphous region is used as the switching phase, rather than traditional crystalline or liquid crystal domains [316]. Based on ultra-high-molecular-weight polyethylene (UHMWPE) with *M*_w_ = 7.0 × 10^6^ g/mol, a material has been created that, after programming at 150 °C and deformation of 1200%, demonstrates a degree of chain orientation of 0.76 in crystalline and 0.20 in amorphous regions. The material exhibits reversible deformation of 13.97% in the range of 30–120 °C without a clearly defined activation point and a record work density of 404.64 J/kg (383.18 kJ/m^3^), which is 47 times higher than that of mammalian skeletal muscle. The practical implementation of the concept included the creation of a biomimetic crawling robot (travel speed 1.07 cm/h with a load of 0.35 g), a precision lifting device, and an adaptive optical system (Figure 9(c1–c3)). This system used a reversible UHMWPE ring as an actuator for a deformable silicone rubber lens (Figure 9(c1,c2)). Owing to the actuator’s reversible change in radius with temperature, the focal length of the lens could be precisely tuned, changing from 647 mm at 30 °C to 314 mm at 120 °C. This resulted in a 101.85% change in image scale, as confirmed by the varying size of a rose observed through the lens (Figure 9(c3)). This methodology opens up new prospects for the development of soft robots and adaptive systems with continuous control based on amorphous phases, overcoming the fundamental limitations of traditional RSMP materials.

## 11. Future Prospects

Recent studies indicate several emerging trends in SMP research [18,317]. SMPs that respond to multifunctional stimulus types are being explored to allow for different actuation types. These systems can be programmed and are attractive for biomedical devices and soft robotic elements where single-stimulus control is insufficient.

Another dominant trend is the use of SMPs in additive manufacturing and 4D printing, an area that has rapidly progressed from proof-of-concept demonstrations to patient-specific and device-oriented designs. Recent studies have reported DLP- and DIW-printed SMP porous architectures based on low molecular weight poly((D,L)-lactide-co-caprolactone) and PCL-based polymers, with precise control over activation temperature and shape recovery being achieved [20,260,288]. These advances enable self-deploying stents, adaptive bone scaffolds, and soft robotic actuators, highlighting 4D printing as a key enabling technology.

To meet the functional demands imposed by these advanced applications, effective control over the activation temperature is a central design challenge, and the choice of tuning strategies inevitably involves inherent trade-offs. Plasticisation using low-molecular-weight additives allows broad and easily adjustable reductions in *T*_g_. However, it may introduce risks associated with additive migration and long-term property instability. In contrast, copolymerisation strategies, such as PLA–PCL systems, offer more stable and intrinsic control over thermal transitions, though with reduced flexibility for post-synthesis tuning.

Biodegradable SMP systems have emerged as a distinct research direction. Several recent studies demonstrate fully or partially bioresorbable SMP-based scaffolds and drug-delivery carriers fabricated from PLA- or PCL-based SMPs, capable of temporary mechanical function followed by controlled degradation. Although still at an early stage, these systems underline a growing interest in transient implants for biomedical intervention, where long-term material persistence is undesirable [206,318,319,320].

Dynamic and self-healing SMP networks, including vitrimer systems and polymers with reversible covalent bonds, have also gained attention. Such materials enable reprogrammable permanent shapes, damage recovery, and extended service life, which is particularly relevant for soft robotic components subjected to cyclic deformation.

Despite these advances, significant challenges remain. Scalability and standardisation continue to limit industrial and clinical adoption, as many SMP systems rely on laboratory-scale synthesis and lack unified testing protocols. Long-term biocompatibility and safety, especially for nanoparticle-filled SMPCs intended for implantation, remain insufficiently explored beyond short-term in vivo studies. In addition, the complexity of multi-stimulus control demands advanced modelling and precise manufacturing to prevent unintended activation or thermal cross-effects. For biodegradable SMPs, achieving a reliable match between degradation kinetics and tissue healing timelines remains a critical materials design challenge. An emphasis on bio-based polymers, green synthesis routes, and clinically relevant activation windows is becoming increasingly evident in the recent literature. Addressing all these issues will be essential for translating the rapidly growing body of SMP research into reliable biomedical devices and next-generation soft robotic systems.

## 12. Conclusions

SMPs have progressed from experimental laboratory materials to versatile smart materials with significant potential in biomedicine and soft robotics. In biomedical applications, the most promising candidates are biodegradable SMPs with activation temperatures tailored to the physiological range of 30–37 °C. SMPs such as PLA, PCL, and their copolymers (e.g., PLCL) provide a well-balanced combination of biodegradability, biocompatibility, and tunable shape-memory performance. To fully exploit these material properties, appropriate fabrication strategies are essential, as biomedical devices require not only biocompatibility but also accurate geometric fidelity, mechanical strength, and structural integrity to maintain functionality under physiological loads. In this context, DLP and DIW additive manufacturing techniques are particularly well-suited for producing patient-specific scaffolds, stents, and membranes with controlled porosity and high shape fidelity, while electrospinning remains an effective approach for fabricating fibrous architectures for wound dressings and neural interfaces.

Building on these materials and processing advances, the focus in soft robotics has shifted toward systems capable of rapid actuation within seconds, high cycling stability, and responsiveness to multiple external stimuli. Among the various activation strategies, magnetoactive SMPCs driven by alternating magnetic fields are especially attractive for wireless actuation and deep-tissue operation due to their high penetration depth. By comparison, photothermally activated SMPCs enable highly localised and precise actuation, although their applicability is constrained by limited optical penetration. Electroactive SMPs incorporating conductive networks offer fast Joule heating and accurate local control, making them suitable for integrated electronic and robotic systems, albeit at the cost of requiring wired connections. Additive manufacturing approaches, including FDM and DLP-based 4D printing, play a critical role in translating these actuation concepts into complex functional components such as grippers, actuators, and adaptive sensors.

Despite significant progress, several limitations still hinder the wider adoption of SMP technologies. Key challenges include low thermal conductivity leading to slow or non-uniform activation, inherent trade-offs between mechanical robustness and shape recovery rate, and the limited availability of long-term in vivo performance data. In addition, the lack of standardised testing protocols complicates comparison between different SMP systems. Addressing these issues will require incremental improvements in material formulations, improved control over filler dispersion and processing, and more systematic preclinical evaluation. Progress in these areas is necessary to move SMPs from laboratory-scale demonstrations toward reliable biomedical devices and soft robotic components for practical applications.

## Figures and Tables

**Table 1:** Mechanical properties and stimuli-responsive behaviour of SMPs.

Fillers	SMP Matrix	Mechanical Properties	External Stimuli	*T*_trans_	*R*_r_ (%)	Ref.
-	Azobenzene-containing polymer network	Modulus: 130 MPa (trans), 23 MPa (cis); Elongation: 160% (trans), 280% (cis)	UV light (365 nm, 40 mW)	*T*_g_ = 42 °C (trans), *T*_g_ = −28 °C (cis)	85	[114]
CNT	Poly(2-hydroxyethyl methacrylate-co-polyethylene glycol diacrylate)	Storage modulus: 4917.2 MPa	NIR light (808 nm laser, 0.67 W/cm^2^)	*T*_g_ = 72.3 °C	98.5	[117]
(MWCNTs-ZnO)@PDA	PLLA	Compressive modulus: 0.9 MPa; Strength: 5 MPa	NIR light (808 nm, 1.1 W/cm^2^)	*T*_g_ = 56–57 °C	100	[118]
Urushiol–Fe	PU	Tensile strength: 17.21 MPa	NIR light (808 nm, 0.5–1.0 W/cm^2^)	*T*_g_ = 37–80 °C	97.8	[125]
Graphene foam	Epoxy resin	Tensile strength: 23.0 MPa	Joule heating (20 V DC)	*T*_g_ = 47.9 °C	100	[138]
Fe_3_O_4_	Polyethylene terephthalate glycol	Tensile strength: 36.24 MPa; Elongation: 12.62%	Magnetic field (coil 30 V, 10 A)	*T*_g_ = 90.8 °C	97.5	[7]
rGO-Fe_3_O_4_	Polyvinyl pyrrolidone/PVA/PEG	Modulus: 1.75 GPa; Tensile strength: 14 MPa; Elongation: 0.05%	Electricity (30 V, 1 A)	*T*_g_ = 55 °C	80	[47]
-	Poly(butanetetrol fumarate)	Elastic Modulus: 26.5 kPa	Water	*T*_g_ = 127.3 °C	95	[150]
GO, CNT	Waterborne epoxy	Storage modulus: 2248 MPa	Thermal heating in water	*T*_g_ = 60.2 °C	98.8	[46]
-	PU	Elastic modulus: 15–153 kPa; Tensile strength: 45–70 kPa; Elongation: 450%	Water	*T*_g_ > 40 °C (dry); *T*_g_ < 37 °C (wet)	100	[151]
-	Cyanate ester/PEG	Tensile strength: 67.64 MPa; Tensile modulus: 1.15 GPa; Max strain: 5.9%	Thermal heating	*T*_g_ = 129.5 °C	100	[152]
Zn	Sulphonated poly(ether ether ketone) (PEEK)	Modulus: 2200 MPa; Yield strength: 61 MPa; Elongation: 15%	Thermal heating	*T*_g_ = 253 °C	99	[153]
-	SMP with silyl ether dynamic covalent linkages	Tensile strength: 82.4 MPa; Modulus: 1863.9 MPa; Elongation: 8.0%	Thermal heating	*T*_g_ = 129.3 °C	99.1	[154]
-	Copolyimide	Storage modulus: 1959 MPa	Thermal heating	*T*_g_ = 196 °C	>96 (stretchable), 100 (deployable)	[155]

## Data Availability

Data are contained within the article.
